# The Navigation Guide—Evidence-Based Medicine Meets Environmental Health: Systematic Review of Human Evidence for PFOA Effects on Fetal Growth

**DOI:** 10.1289/ehp.1307893

**Published:** 2014-06-25

**Authors:** Paula I. Johnson, Patrice Sutton, Dylan S. Atchley, Erica Koustas, Juleen Lam, Saunak Sen, Karen A. Robinson, Daniel A. Axelrad, Tracey J. Woodruff

**Affiliations:** 1 Program on Reproductive Health and the Environment, University of California, San Francisco, Oakland, California, USA; 2 Oak Ridge Institute for Science and Education (ORISE) Postdoctoral Fellowship, National Center for Environmental Economics, Office of Policy, U.S. Environmental Protection Agency, Washington, DC, USA; 3 Department of Epidemiology and Biostatistics, University of California, San Francisco, San Francisco, California, USA; 4 Department of Medicine,; 5 Department of Epidemiology, and; 6 Department of Health Policy & Management, Johns Hopkins University, Baltimore, Maryland, USA; 7 National Center for Environmental Economics, Office of Policy, U.S. Environmental Protection Agency, Washington, DC, USA

## Abstract

Background: The Navigation Guide methodology was developed to meet the need for a robust method of systematic and transparent research synthesis in environmental health science. We conducted a case study systematic review to support proof of concept of the method.

Objective: We applied the Navigation Guide systematic review methodology to determine whether developmental exposure to perfluorooctanoic acid (PFOA) affects fetal growth in humans.

Methods: We applied the first 3 steps of the Navigation Guide methodology to human epidemiological data: *1*) specify the study question, *2*) select the evidence, and *3*) rate the quality and strength of the evidence. We developed a protocol, conducted a comprehensive search of the literature, and identified relevant studies using prespecified criteria. We evaluated each study for risk of bias and conducted meta-analyses on a subset of studies. We rated quality and strength of the entire body of human evidence.

Results: We identified 18 human studies that met our inclusion criteria, and 9 of these were combined through meta-analysis. Through meta-analysis, we estimated that a 1-ng/mL increase in serum or plasma PFOA was associated with a –18.9 g (95% CI: –29.8, –7.9) difference in birth weight. We concluded that the risk of bias across studies was low, and we assigned a “moderate” quality rating to the overall body of human evidence.

Conclusion: On the basis of this first application of the Navigation Guide systematic review methodology, we concluded that there is “sufficient” human evidence that developmental exposure to PFOA reduces fetal growth.

Citation: Johnson PI, Sutton P, Atchley DS, Koustas E, Lam J, Sen S, Robinson KA, Axelrad DA, Woodruff TJ. 2014. The Navigation Guide—evidence-based medicine meets environmental health: systematic review of human evidence for PFOA effects on fetal growth. Environ Health Perspect 122:1028–1039; http://dx.doi.org/10.1289/ehp.1307893

## Introduction

Synthesizing environmental health research from multiple streams of evidence is critical to translating the science into improved health outcomes. Robust, systematic, and transparent methods of research synthesis are an identified need in environmental health (National Research Council 2011). Such methods already exist to evaluate clinical evidence (GRADE Working Group 2012; Higgins and Green 2011) and include steps such as developing a prespecified protocol, a comprehensive search, and rating the quality of the evidence. Although methods of synthesizing clinical research are primarily applied to randomized controlled clinical trials, the evidence streams for environmental health science are different. The Navigation Guide systematic review methodology was developed to apply best practices in research synthesis in clinical medicine and environmental health to the evidence streams common in environmental health science (i.e., experimental toxicological studies and observational human studies) in order to reach an overall conclusion about the strength of evidence (Woodruff et al. 2011a). Additional background on the Navigation Guide is given in a companion commentary (Woodruff and Sutton 2014).

We undertook a case study to apply the Navigation Guide methodology. For this first case study, we evaluated the evidence for the effects of exposure to perfluorooctanoic acid (PFOA) on fetal growth. PFOA has been used for > 50 years in the manufacture of fluoropolymers used in industrial applications and consumer products to impart certain characteristics, such as fire and stain resistance [Prevedouros et al. 2006; U.S. Environmental Protection Agency (EPA) 2012]. We selected PFOA for evaluation based on pervasive human exposure and the evidence of associations with fetal growth (Agency for Toxic Substances and Disease Registry 2009; Apelberg et al. 2007; Fei et al. 2007, 2008; Kato et al. 2011; U.S. EPA 2012). In addition, there have been inconsistent conclusions about the evidence of an association between PFOA and fetal growth (DeWitt et al. 2009; Hekster et al. 2003; Jensen and Leffers 2008; Kennedy et al. 2004; Kudo and Kawashima 2003; Lau et al. 2004, 2007; Lindstrom et al. 2011; Olsen et al. 2009; Post et al. 2012; Steenland et al. 2010; White et al. 2011).

In this review, we evaluate the human epidemiologic evidence relating PFOA exposure to fetal growth using the Navigation Guide systematic review approach. The results of applying the Navigation Guide methodology to the nonhuman evidence are presented in another review (Koustas et al. 2014), and the results of applying the Navigation Guide methodology to integrate the human and nonhuman evidence streams are presented in a third review (Lam et al. 2014).

## Methods

We assembled a review team with expertise in the fields of systematic review, environmental health, epidemiology, biology, statistics, and risk assessment to develop a a prespecified protocol for conducting the systematic review [University of California, San Francisco (UCSF) Program on Reproductive Health and the Environment 2013]. More information about the review team is available in the companion commentary by Woodruff and Sutton (2014).

### Step 1. Specify the Study Question

Our objective was to answer the question: “Does fetal developmental exposure to PFOA affect fetal growth in humans?” We developed a PECO (participants, exposure, comparator, and outcomes) statement, which is used as an aid to developing an answerable question (Higgins and Green 2011). Our PECO statement included the following:

Participants: humans who are studied during the reproductive/developmental time period (before and/or during pregnancy or development)
Exposure: exposure to PFOA (CAS# 335-67-1) or its salts during the time before pregnancy and/or during pregnancy for females or directly to fetuses
Comparators: humans exposed to lower levels of PFOA than the more highly exposed humans (i.e., a comparison across a range of exposures)
Outcomes: effects on fetal growth, birth weight, and/or other measures of size, such as length.

### Step 2. Select the Evidence

*Search methods.* We developed search terms to identify relevant literature by using the medical subject headings (MeSH) in PubMed (http://www.ncbi.nlm.nih.gov/pubmed) and other key words for five articles known to us and that we judged to be relevant to our study question. Our search was not limited by language or publication date. The search terms for each database, which include terms related to the exposure, the outcome, and the human subjects, are provided in Supplemental Material, Table S1. We searched PubMed (30 April 2012), Embase (http://www.elsevier.com/online-tools/embase; 7 May 2012), Web of Science (http://apps.webofknowledge.com/; 11 May 2012), and other databases (23–27 April 2012). The specific databases searched and numbers of records retrieved are provided in Supplemental Material, Table S2. We also hand searched the reference lists of all included studies and used Web of Science to search for articles that cited the included studies.

*Study selection criteria.* We selected studies in which human exposure to PFOA was measured or estimated, and associations with fetal growth were evaluated. We did not require fetal growth to be the main outcome of the study. We screened studies for inclusion using a structured form in DistillerSR (Evidence Partners; http://www.systematic-review.net). Two review authors (P.I.J. and D.S.A.) independently conducted a title and abstract screen of the search results to determine whether a reference met the inclusion criteria; studies that were not excluded based on the title and abstract were screened through a full-text review. A third author (P.S.) screened 5% of the search results at the title/abstract and full-text stages for quality assurance. Following the screening and in the case of discrepant results between reviewers, the initial two reviewers (P.I.J. and D.S.A.) discussed each discrepancy and brought in the third reviewer (P.S.) if necessary to discuss and decide on the status (include/exclude) of each discrepancy.

We excluded studies if

The article did not contain original data or observations
Study subjects were not humans
Exposure to PFOA was not measured in, or estimated for, the study subjects
PFOA exposure was not measured or estimated during the reproductive/developmental time period (any time before or during pregnancy for women, or directly in fetuses, including cord blood)
Fetal or infant growth or birth weight was not measured.

*Data collection.* Two review authors (P.I.J. and D.S.A.) worked together to extract the data from all of the included articles. We compared all of the extracted data with the same data that was independently extracted by a third researcher (J. Pan; UCSF) for quality assurance and quality control. We planned to discuss any discrepancies among the three extractors to come to a consensus. We collected details of the study characteristics, exposure assessment, outcome measurements, and information used to assess risk of bias using a structured form in DistillerSR; we created this form by combining aspects of existing criteria and checklists (Guyatt et al. 2011b; Higgins and Deeks 2011; von Elm et al. 2008). We contacted study authors to obtain data needed for the analysis that were not reported in the published articles.

### Step 3. Rate the Quality and Strength of the Evidence

**Figure 1:**
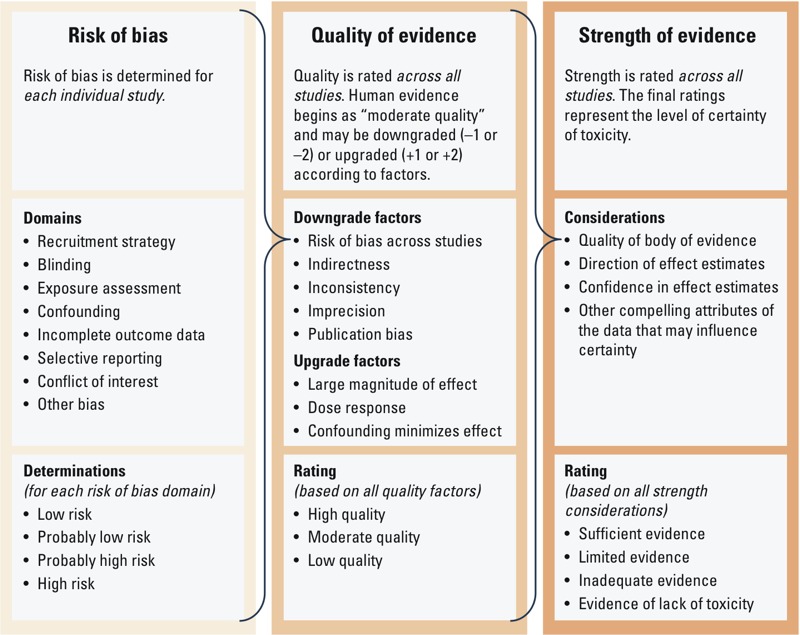
Overview of Navigation Guide systematic review methodology used for rating the quality and strength of the human evidence.

We rated the quality and strength of the evidence by *a*) assessing “risk of bias,” defined as characteristics of a study that can introduce a systematic error in the magnitude or direction of the results of the study (Higgins and Green 2011), for each included study; *b*) rating the quality of the evidence across all studies; and *c*) rating the strength, or certainty, of the evidence across all studies (Figure 1).

*Assessing the risk of bias for each included study.* We assessed risk of bias for each included study using an instrument we developed by adapting existing risk of bias guidance used to evaluate human studies in the clinical sciences: the Cochrane Collaboration’s Risk of Bias tool (Higgins and Green 2011) and the Agency for Healthcare Research and Quality’s criteria (Viswanathan et al. 2012). We also included the funding source and declared conflicts of interest as a potential source of bias based on empirical data from studies conducted on pharmacological treatments that reported evidence of bias associated with funding source (Krauth et al. 2013; Lundh et al. 2012). We considered whether the study received “support from a company, study author, or other entity having a financial interest in the outcome of the study” (see Supplemental Material, “Instructions for Making Risk of Bias Determinations”). Although we refer generally to this risk of bias domain as “conflicts of interest,” we only assessed competing financial interests in this case study.

We assigned each risk of bias domain as “low risk,” “probably low risk,” “probably high risk,” “high risk,” or “not applicable” (risk of bias area not applicable to the study). Our protocol provided specific instructions for each classification to help ensure consistent interpretation. The specific risk of bias instrument is provided in Supplemental Material, “Instructions for Making Risk of Bias Determinations.” A summary of the criteria for “low risk” of bias for each domain is outlined in Table 1. Two review authors (P.I.J. and D.S.A.) independently made and documented risk of bias determinations for each study across all domains. When these two authors could not reach consensus on a risk of bias domain, two other authors (P.S. and J.L.) reviewed the results. If upon further discussion the four authors were unable to reach agreement on a risk of bias determination for a particular domain, the more conservative judgment was selected (e.g., if one reviewer made a judgment of “low risk” and the other made a judgment of “probably low risk,” the “probably low risk” judgment was used).

**Table 1:** Summary of risk of bias designations for individual human studies.

Risk of bias domain	Low risk of bias designation^*a*^
Recruitment strategy	Participant recruitment protects against selection bias
Blinding	Knowledge of exposure is prevented when assessing outcome
Exposure assessment	Risk of exposure misclassification is minimized through validated methods
Confounding	Important potential confounders were appropriately accounted for
Incomplete outcome data	Any missing outcome data is not likely to introduce bias
Selective outcome reporting	All outcomes specified in methods have been reported
Conflict of interest	Study free of support from individual or entity having financial interest in outcome of study
Other bias	Study appears to be free of other sources of bias
^***a***^Complete criteria for each risk of bias designation are provided in Supplemental Material, “Instructions for Making Risk of Bias Determinations.”	

In addition to the instructions in the protocol, we made the following decisions for rating the blinding and confounding risk of bias domains. We judged a study to be low risk of bias for blinding when it described that specimen samples were coded or otherwise blinded. We judged a study to be probably low risk of bias when it described only partial blinding or described methods that would have effectively blinded investigators to the exposure and outcome groups (e.g., exposure was measured separately, and birth weight was obtained from a hospital record). Our criterion for designating low risk of bias for confounding required that studies account for only two potential confounders, those we deemed the main “important” confounders. Based on existing data and *a priori* knowledge of the exposure and outcome of interest in all the studies, we decided that maternal age and gestational age were the main “important” potential confounders for all the included studies. Maternal age and gestational age may be correlated with PFOA exposure and fetal growth (Fei et al. 2007; Halldorsson et al. 2012; Ode et al. 2013). We considered studies “low risk” of bias for confounding if the study authors accounted for both maternal age and gestational age in their design or analysis or if they reported that either confounder did not influence associations between PFOA and the outcome being asessed. We considered studies high risk of bias if the study authors did not account for either maternal age or gestational age, and probably high risk if they did not account for gestational age in the data analysis but restricted the analysis of birth weight to term births due to the potential for residual confounding by gestational age among term births.

*Data evaluation and meta-analysis.* We assessed the following study characteristics to determine whether studies were combinable in a meta-analysis: measure of fetal growth outcome, study design, exposure assessment, and data analysis. We compared different measures of exposure, such as cord or maternal serum, to determine whether they were comparable. Biomarkers and timing of PFOA exposure assessments differed across studies, that is, PFOA was measured at different times during pregnancy in cord serum, maternal serum, and maternal plasma. Despite these differences, there was evidence of high correlation of PFOA concentrations between these matrices within the same populations: between cord and maternal serum at birth (Fromme et al. 2010; Kim SK et al. 2011; Monroy et al. 2008); between cord and second or third trimester maternal serum or plasma (Fei et al. 2007; Kim S et al. 2011); and between first and second trimester maternal plasma (Fei et al. 2007). We combined studies in the meta-analyses with different measures of PFOA based on this concordance. We also assessed the following study design and data analysis parameters to determine which studies were combinable: the exposure and outcome statistic (continuous, dichotomous, or other scale), whether studies measured actual birth weights or only recorded whether the birth weight was “low” (i.e., < 2,500 g) and which variable was the dependent or independent variable in models of estimated effects. We contacted study authors to request information needed for meta-analysis. We requested linear regression model coefficients for the association between a 1-ng/mL increase in PFOA (modeled as an untransformed continuous variable) and each outcome from authors if they were not reported, or, if less burdensome for authors, the raw data needed to calculate the estimated difference in birth weight. When raw data were provided, we used linear regression models to calculate estimates of change in birth weight per nanogram per milliliter of serum PFOA. We evaluated potential confounders as provided by study authors, one at a time compared with an unadjusted model. We adjusted the regression models by the covariates provided by the study authors if inclusion of the covariate changed the estimate by > 10% compared with the unadjusted model.

We conducted computations for linear regressions, meta-analyses and heterogeneity statistics using STATA, version 12.1 (StataCorp LP). We used the “metaan” command in STATA with the DerSimonian-Laird random-effects method for all meta-analyses to account for potential heterogeneity across studies (DerSimonian and Laird 1986). We used estimates of association between PFOA and fetal growth and the standard error from each study to calculate an overall effect estimate for the following fetal growth measures at birth: weight, length, head circumference, and ponderal index (birth weight divided by length cubed, multiplied by 100).

To test statistical heterogeneity across the study estimates, we estimated the variance corresponding to the between-study variability, and tested the null hypothesis that the between-study variability was absent. We used the Cochran’s *Q* statistic for this test, which follows a chi-square distribution with *n* – 1 degrees of freedom (df), where *n* is the number of studies. We considered a *p*-value of ≤ 0.05 statistically significant. We calculated and evaluated the *I*^2^ test statistic [*I*^2^ = 100 × (*Q –* df)/*Q*], which is an estimate of the percentage of the variability among study estimates that is due to heterogeneity rather than chance (Higgins and Green 2011). We conducted sensitivity analyses to determine the effect of including estimates from studies with differing characteristics or high risk of bias.

*Rating the quality of evidence across studies.* The possible ratings for the overall quality of the body of evidence were ”high,” ”moderate,” or ”low.” Following the approach established by the Grading of Recommendations Assessment Development and Evaluation (GRADE) method used in the clinical sciences for making evidence-based decisions for medical interventions (GRADE Working Group 2012), we determined the final rating by assigning a prespecified initial quality rating to the body of evidence, and then considering adjustments (“downgrades” or “upgrades”) to the quality rating based on the characteristics of the studies constituting the body of evidence (Balshem et al. 2011). GRADE guidelines, developed for clinical interventions, assign experimental human studies an initial rating of “high” and observational studies an initial rating of “low” quality (Balshem et al. 2011). However, there is variability in the quality of studies, and not all observational studies are low quality (Viswanathan et al. 2012). In environmental health, human observational data are usually the most directly applicable data available for decision making because ethical considerations virtually preclude human randomized controlled trials (RCTs) in environmental health. We began by rating human observational studies at “moderate” quality to capture both the limitations of observational data and their recognized value in assessing associations between exposure and health outcomes and disease etiology in environmental and clinical sciences (Woodruff and Sutton 2014). When available, experimental human data or “natural experiments” may be considered as “high” quality data if they are comparable to RCTs; however, this was not relevant to this case study, and thus the criteria for determining comparability with RCTs is not yet developed.

We assessed the overall body of human evidence for downgrading and upgrading the prespecified “moderate” quality rating based on eight factors (Table 2). In addition, because we were primarily concerned with underestimating true positive associations in evaluating the potential for publication bias, we therefore considered *a*) whether the body of evidence was dominated by early studies with negative associations, particularly if the studies were small; *b*) whether studies were uniformly small; *c*) if there were enough studies to conduct an examination of patterns of study results (e.g., funnel plots) that might suggest publication bias; *d*) if we obtained unpublished studies with results that differed from published studies; or *e*) whether a comprehensive literature search was performed. Possible ratings were 0 (no change from “moderate” quality), –1 (one-level downgrade), –2 (two-level downgrade); +1 (one-level upgrade), or +2 (two-level upgrade). The review authors independently evaluated the quality of the evidence and then compared their ratings and rationale for each quality factor. We resolved discrepancies between individual author ratings through discussion until consensus was reached. We were conservative in our judgments of downgrading or upgrading the evidence, consistent with the GRADE approach (i.e., we required compelling rationale) (Guyatt et al. 2011a). We recorded the collective rationale for decisions on the eight factors and for the final rating.

**Table 2:** Factors for evaluating the quality of the body of human evidence.

Evaluation factors	Summary of criteria
Downgrading factors
Risk of bias	Study limitations include a substantial risk of bias across the body of evidence.
Indirectness	Evidence was not directly comparable to the question of interest [i.e., population, exposure, comparator, outcome (PECO)].
Inconsistency	Estimates of effect in similar populations were widely different (heterogeneity or variability in results).
Imprecision	Studies included few participants and few events (wide CIs).
Publication bias	Studies were missing from body of evidence, resulting in an over- or underestimate of true effects from exposure.
Upgrading factors
Large magnitude of effect	The rating was upgraded if modeling suggested that confounding alone was unlikely to explain associations that were judged to be of large magnitude.
Dose response	The rating was upgraded if the relationship between dose and response in one or multiple studies and/or the dose response across studies were consistent.
Confounding minimizes effect	The rating was upgraded if the consideration of all plausible residual confounders or biases would underestimate the effect or suggest a spurious effect when results show no effect.

*Rating the strength of the evidence across studies.* Rating the strength of the evidence across the human studies summarizes the human evidence; this summary will allow for the integration of human and nonhuman streams of evidence, ultimately leading to a concise statement about a chemical’s toxicity (Woodruff et al. 2011a).

We rated the overall strength of the body of human evidence based on a combination of four considerations: *a*) quality of the body of evidence (i.e., the rating from the previous step); *b*) direction of the effect estimate; *c*) confidence in the effect estimate (likelihood that a new study would change our conclusion); and *d*) other compelling attributes of the data that may influence certainty (Figure 1). We compared the results of rating the strength of the human evidence to the definitions specified in the Navigation Guide for “sufficient evidence of toxicity,” “limited evidence of toxicity,” ”inadequate evidence of toxicity,” or “evidence of lack of toxicity” (Table 3). We based the rating categories for the strength of the evidence on those used by the International Agency for Research on Cancer (IARC 2006). We used criteria and considerations used by IARC (2006), the U.S. Preventive Services Task Force (Sawaya et al. 2007), and the U.S. EPA (1991, 1996) for the type of evidence considered for each of these strength of evidence categories. Review authors independently evaluated the strength of the evidence according to the same four considerations and compared their evaluations, resolved discrepancies by discussion, and recorded the collective rationale for decisions.

**Table 3:** Strength of evidence definitions for human evidence.^*a*^

Strength rating	Definition
Sufficient evidence of toxicity	A positive relationship is observed between exposure and outcome, where chance, bias, and confounding can be ruled out with reasonable confidence. The available evidence includes results from one or more well-designed, well-conducted studies, and the conclusion is “unlikely to be strongly affected by the results of future studies.”^*b*^
Limited evidence of toxicity	A positive relationship is observed between exposure and outcome, where chance, bias, and confounding cannot be ruled out with reasonable confidence. Confidence in the relationship is constrained by factors such as “the number, size, or quality of individual studies” or “inconsistency of findings across individual studies.”^*b*^ As more information becomes available, the observed effect could change, and this change may be large enough to alter the conclusion.
Inadequate evidence of toxicity	“The available evidence is insufficient to assess effects” of the exposure. The evidence is insufficient because of “the limited number or size of studies,” low quality of individual studies, or “inconsistency of findings across individual studies.”^*b*^ More information may allow an assessment of effects.
Evidence of lack of toxicity	No relationship is observed between exposure and outcome; and chance, bias, and confounding can be ruled out with reasonable confidence. The available evidence includes consistent results from more than one well-designed, well-conducted study at the full range of exposure levels that humans are known to encounter, and the conclusion is unlikely to be strongly affected by the results of future studies.^*b*^ The conclusion is limited to the age at exposure and/or other conditions and levels of exposure studied.
^***a***^The Navigation Guide rates the quality and strength of evidence of human and non­human evidence streams separately as “sufficient,” “limited,” “inadequate,” or “evidence of lack of toxicity,” and then these two ratings are combined to produce one of five possible statements about the overall strength of the evidence of a chemical’s reproductive/developmental toxicity. The methodology is adapted from the criteria used by IARC to categorize the carcinogenicity of substances (IARC 2006) except as noted. ^***b***^Language for the definitions of the rating categories were either from or adapted from descriptions of levels of certainty provided by the U.S. Preventive Services Task Force levels of certainty regarding net benefit (Sawaya et al. 2007).	

## Results

**Figure 2:**
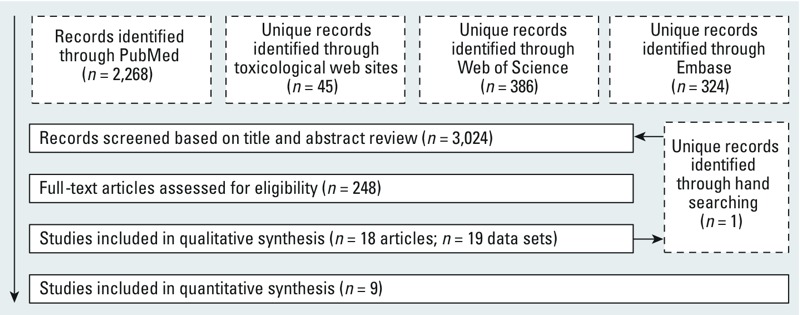
Flowchart showing the literature search and screening process. The primary goal of our search was to obtain comprehensive results; therefore, our search was not limited by language or publication date. The search terms for each database are provided in Supplemental Material, Table S1.

*Included studies*. Our search retrieved a total of 3,023 unique records, of which 17 articles met the inclusion criteria (Figure 2). One of the 17 articles contained the results of two separate data sets (Savitz et al. 2012b), and hand searching the reference lists of the 17 included articles identified 1 additional study not yet indexed in the databases searched. Therefore, we included a total of 19 data sets in our analysis (Table 4). The studies covered the years 1988–2009, included populations located in nine countries, and ranged from 17 to 11,737 study subjects (Table 4).

**Table 4:** Summary of study characteristics evaluating developmental exposure to PFOA and fetal growth in human observational studies.

Source	Fetal growth measures^*a*^	Births (*n*)	Location	Study period	Sample matrix	Measurement timing	Median PFOA (ng/mL)	Reason for omission from meta-analysis
Studies included in meta-analyses
Apelberg et al. 2007	BW, L, HC, PI	293	Baltimore, MD (USA)	2004–2005	Cord serum	Birth	1.6	NA
Chen et al. 2012	BW, L, HC, PI	429	Taipei, Taiwan	2004	Cord serum	Birth	1.7	NA
Fei et al. 2007^*b*^	BW	1,400	Denmark	1996–2003	Maternal plasma	1st trimester	5.2	NA
Fei et al. 2008^*b*^	L, HC, PI,^*c *^AC^*d*^	1,400	Denmark	1996–2003	Maternal plasma	1st trimester	5.2	NA
Fromme et al. 2010	BW	33	Munich, Germany	2007–2008	Cord serum	Birth	1.4	NA
Hamm et al. 2009	BW, SGA^*d*^	252	Edmonton, Canada	2005–2006	Maternal serum	15–16 weeks	1.5	NA
Kim S et al. 2011	BW	43	South Korea	2008–2009	Cord serum	Birth	1.2	NA
Maisonet et al. 2012	BW, L, PI	422	Great Britain	1991–1992	Maternal serum	15 weeks (median)	3.7	NA
Washino et al. 2009	BW, L, HC, PI, CC^*d *^	428	Sapporo, Japan	2002–2005	Maternal serum	23–35 weeks	1.3	NA
Whitworth et al. 2012	BW, SGA,^*d*^ LGA^*d*^	849	Norway	2003–2004	Maternal plasma	17 weeks to term	2.2	NA
Studies excluded from meta-analysis
Arbuckle et al. 2012	BW	100	Ottawa, Ontario, Canada	2005–2008	Cord serum	Birth	1.6	BW is not the dependent model variable^*e*^
Halldorsson et al. 2012	BW	665	Aarhus, Denmark	1988–1989	Maternal serum	30 weeks	3.7	Only mean BW per PFOA quartile given
Kim SK et al. 2011	BW	17	Seoul, Korea	2007	Cord serum	Birth	1.1	Only PFOA–BW correlation given
Monroy et al. 2008	BW	101	Ontario, Canada	2004–2005	Cord serum	Birth	1.6	BW is not the dependent model variable^*e*^
Nolan et al. 2009	BW	1,555	Ohio (USA)	2002–2005	Water service area	Preconception or during pregnancy	NA	Categorical ecological exposure^*f *^
Savitz et al. 2012a	BW	10,189	Ohio and West Virginia (USA)	1990–2006	Retrospectively modeled maternal serum	During pregnancy	6–15.9	High risk of bias for exposure assessment; only dichotomous outcome of low BW^*f *^
Savitz et al. 2012b (study I)	BW	8,253	Ohio and West Virginia (USA)	1990–2004	Retrospectively modeled maternal serum	During pregnancy	7.7	High risk of bias for exposure assessment; (included estimate from Study II in sensitivity meta-analysis)^*f *^
Savitz et al. 2012b (study II)	BW	4,547	Ohio and West Virginia (USA)	1990–2004	Retrospectively modeled maternal serum	During pregnancy	7.2–18.3	High risk of bias for exposure assessment; (Included in sensitivity meta-analysis)^*f *^
Stein et al. 2009	BW	1,589	Ohio and West Virginia (USA)	2000–2006	Maternal serum	Up to 5 years postnatal	21.2	Only dichotomous outcome of low BW^*f *^
Abbreviations: AC, abdominal circumference; BW, birth weight; CC, chest circumference; HC, head circumference; L, length; LGA, large for gestational age; NA, not applicable; PI, ponderal index; SGA, small for gestational age. ^***a***^All fetal growth measures at birth. ^***b***^Fei et al. (2007) and Fei et al. (2008) are studies of the same population. ^***c***^Because the analysis of PI was stratified, it was not combined in the meta-analysis of PI. ^***d***^Only two studies measured SGA, and only one study each measured AC, CC or LGA; no meta-analysis was conducted for these measures. ^***e***^Serum PFOA was the outcome variable estimated in the model. ^***f***^Because the studies of Nolan et al. (2009), Savitz et al. (2012a, 2012b), and Stein et al. (2009) were conducted in the same geographical area, participants may overlap; therefore, we did not consider these studies for simultaneous inclusion in meta-analysis.								

**Figure 3:**
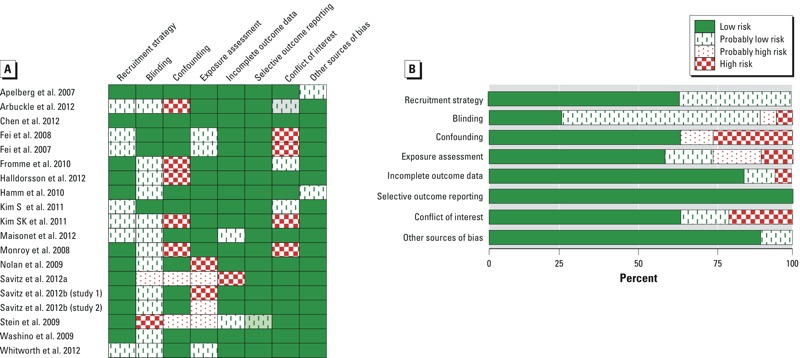
Summary of the risk of bias judgments (low, probably low, probably high, and high risk) for each included human study (*A*) and (*B*) given as percentages across all included human studies. Risk of bias designations for individual studies are assigned according to criteria provided in Supplemental Material, “Instructions for Making Risk of Bias Determinations.”

*Risk of bias assessment for individual studies*. We concluded that there was generally low risk of bias across the 19 data sets (Figure 3A). According to the Navigation Guide criteria, we identified confounding, exposure assessment, and conflict of interest as the most common types of risk of bias (Figure 3B). Although we considered risk of bias separately for each outcome (birth weight and other fetal growth measures), the results did not differ with respect to outcome, so only one summary is presented in Figure 3 (see also Supplemental Material, Tables S3–S21). One exception was Maisonet et al. (2012) (see Supplemental Material, Table S13), for which we designated a higher risk of bias for fetal growth measures other than birth weight because of a large amount of missing data for outcomes other than birth weight in that study.

**Figure 4:**
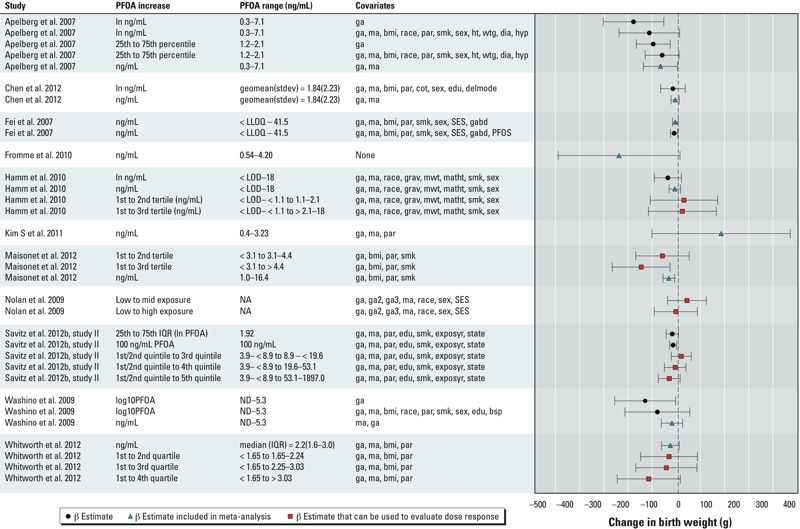
Summary of data extracted from all studies of PFOA exposure that included continuous outcome of birth weight. The PFOA increase is the exposure contrast being compared in each study. Squares represent data for which there was an exposure gradient that can be evaluated in considering dose response in upgrading the quality of the evidence. Error bars indicate 95% CIs. Savitz et al. (2012b) presented additional alternative estimates based on different modeling assumptions that are not included here due to space limitations. Covariate abbreviations: bmi, body mass index; bsp, blood sampling period; cot, serum cotinine; delmode, delivery mode; dia, diabetes; edu, maternal education level; exposyr, year of exposure estimate; ga, gestational age; gabd, gestational age at blood draw; geomean(stdev), geometric mean (geometric SD); grav, gravidity; ma, maternal age; ht, maternal height; hyp, hypertension; mwt, maternal prepregnancy weight; NA, not applicable: ND, not detected; par, parity; PFOS, serum perfluoro­octane sulfonic acid; SES, socioeconomic status; sex, infant sex; smk, smoking status; state, state of residence; wtg, maternal weight gain during pregnancy. This figure was created using Meta Data Viewer (http://ntp.niehs.nih.gov/help/browsers/metadata/index.html) (Boyles et al. 2011).

*Statistical analysis*. We found no discrepancies in the data with respect to different data extractors. We made 13 requests to study authors for additional data for our meta-analysis. Six study authors responded; 3 provided the requested statistics and 3 provided individual-level data from which we calculated the summary statistics. We plotted continuous effect estimates (11 studies) to visually assess the range, precision, and dose–response data for evaluating the relationship between PFOA and birth weight (Figure 4). A summary plot of all odds ratio estimates for low birth weight (< 2,500 g) is presented in Supplemental Material, Figure S1.

**Figure 5:**
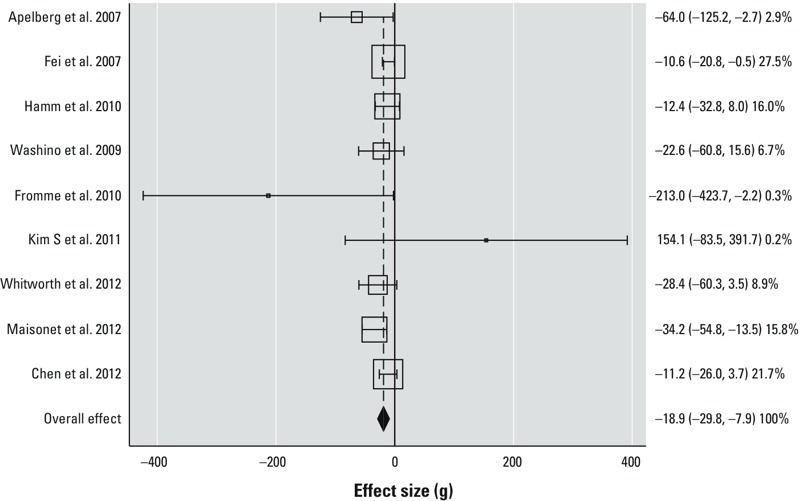
Results of meta-analysis for birth weight (*n *= 9 studies, 4,149 births) shown as effect estimates [change in birth weight in grams per nanogram of PFOA per milliliter of serum or plasma (95% CIs)]. The percentages are weightings of the individual studies in the meta-analysis according to the inverse of the variance, and the sizes of the boxes are scaled accordingly. The dashed line indicates the overall effect estimate derived from the DerSimonian-Laird random effects meta-analysis, and the diamond indicates the 95% CI of the overall effect estimate. Heterogeneity statistics: Cochran’s *Q *= 12.92; *p *= 0.12; *I*^2^ = 38%. Estimates were adjusted as follows: Apelberg et al. (2007): maternal age and gestational age; Fei et al. (2007): maternal age, gestational age, quadratic gestational age, infant sex, socio-occupational status, parity, smoking, pre­pregnancy body mass index, and gestational week at blood draw; Hamm et al. (2010): maternal age, gestational age, race, gravidity, maternal pre­pregnancy weight, maternal height, smoking status, and infant sex; Washino et al. (2009): maternal age and gestational age; Fromme et al. (2010): unadjusted; Kim S et al. (2011): maternal age, gestational age, and parity; Whitworth et al. (2012): maternal age, gestational age, prepregnancy body mass index, and parity; Maisonet et al. (2012): smoking, prepregnancy body mass index, previous live birth, and gestational age; Chen et al. (2012): maternal age and gestational age.

We combined data from 10 studies in the meta-analyses of the association between PFOA exposure and measures of fetal growth. Within the 10 studies, there were 9 data sets on birth weight, 5 data sets on length, 4 data sets on ponderal index, and 4 data sets on chest circumference. The studies that were not included in the meta-analyses (*n* = 9; Table 4) generally reported a statistical estimate that was not combinable with the others. The majority of the studies reported a continuous regression estimate with PFOA as the independent variable and fetal growth as the dependent variable. If a study reported an alternate statistic such as an odds ratio, mean, or correlation coefficient and we were unable to obtain data from the authors, then the study could not be combined with the majority of studies in the meta-analysis (Table 4). From the meta-analysis of 9 studies (4,149 births) of birth weight, we found an overall estimate of –18.9 [95% confidence interval (CI): –29.8, –7.9] g birth weight/ng/mL increase in serum or plasma PFOA (Figure 5, Table 5). We did not find a high level of heterogeneity among the studies in this meta-analysis (Cochran’s *Q* = 12.92; *p* = 0.12; *I*^2^ = 38%).

**Table 5:** Summary of meta-analyses for associations of fetal growth measures with serum or plasma PFOA.

Fetal growth measure	No. of studies	No. of births	Effect estimate from meta-analysis [per ng/mL (95% CI)]	Cochran’s *Q*	*p*-Value for heterogeneity	*I*^2^ (%)
Birth weight (g)	9^*a*^	4,149	–18.9 (–29.8, –7.9)	12.92	0.12	38
Birth weight (g), sensitivity analysis	10^*b*^	8,501	–15.4 (–26.5, –4.3)	31.91	0	72
Length (cm)	5^*c*^	2,853	–0.06 (–0.09, –0.02)	3.03	0.55	0
Ponderal index^*d*^	4^*e*^	1,510	–0.01 (–0.03, 0.01)	8.03	0.05	63
Head circumference (cm)	4^*f*^	2,497	–0.03 (–0.08, 0.01)	4.05	0.26	26
Individual study estimates for outcomes other than birth weight are provided in Supplemental Material, Tables S22–S24. ^***a***^Meta-analysis (*n* = 9 birth weight studies) includes Apelberg et al. (2007), Chen et al. (2012), Fei et al. (2007), Fromme et al. (2010), Hamm et al. (2010), Kim S et al. (2011), Maisonet et al. (2012), Washino et al. (2009), and Whitworth et al. (2012). ^***b***^Sensitivity analysis: Meta-analysis (*n* = 10 birth weight studies) includes the same studies as included in the 9-study meta-analysis plus an additional estimate from Savitz et al. (2012b) (high risk of bias for exposure assessment). ^***c***^Meta-analysis for length includes Apelberg et al. (2007), Fei et al. (2008), Chen et al. (2012), Maisonet et al. (2012), and Washino et al. (2009). ^***d***^Ponderal index equals birth weight divided by length cubed, multiplied by 100. ^***e***^Meta-analysis for ponderal index includes Apelberg et al. (2007), Chen et al. (2012), Maisonet et al. (2012), and Washino et al. (2009). ^***f***^Meta-analysis for head circumference includes Apelberg et al. (2007), Fei et al. (2008), Chen et al. (2012), and Washino et al. (2009).						

We judged the study of Savitz et al. (2012b) to have “probably high” risk of bias for the exposure assessment domain based on its retrospectively modeled maternal serum PFOA (see Supplemental Material, Table S18). However, because this judgment fell within the uncertain (“probably”) area and because others may make a different judgment about the risk of bias, we also conducted the meta-analysis including this study (Table 5). The addition of Savitz et al.’s estimate [from study II, Bayesian calibrated estimate of –0.185 g/ng/mL (95% CI: –0.313, –0.058)] to the meta-analysis did not change the direction of the overall association but reduced its magnitude from an estimated 18.9-g reduction in birth weight/ng/mL serum or plasma PFOA to a 15.4-g reduction (95% CI: –26.5, –4.3), and increased the heterogeneity (from an *I*^2^ of 38% to 72%) (Table 5).

Only one study that we included in the meta-analysis for birth weight was assigned a high risk of bias for confounding (Fromme et al. 2010). This study was small and contributed little weight (< 1%) to the overall effect estimate. Omitting the study of Fromme et al. (2010) from the meta-analysis reduced the magnitude of the association from an estimated 18.9-g reduction in birth weight/ng/mL serum or plasma PFOA to a 17.4-g reduction (95% CI: –26.8, –8.0) and reduced the heterogeneity (from an *I*^2^ of 38% to 27%). Fei et al. (2007) was the only study that we included in the meta-analysis that was assigned a high risk of bias for conflict of interest. Omitting that study increased the magnitude of the association from an estimated 18.9-g reduction in birth weight/ng/mL serum or plasma PFOA to a 22.7-g reduction (95% CI: –36.9, –8.4) and did not change the heterogeneity.

We found through meta-analysis that PFOA exposure was also associated with lower values of other fetal growth measures at birth (Table 5). A 1-ng/mL increase in serum or plasma PFOA was associated with a –0.1 (95% CI: –0.1, –0.02) cm change in birth length, a –0.01 (95% CI: –0.03, 0.01) change in ponderal index, and a –0.03 (95% CI: –0.1, 0.01) cm change in head circumference. Individual study estimates included in these analyses, and weights assigned to each, are provided in Supplemental Material, Tables S22–S24.

We explored the potential effect that a new study might have on our meta-analysis of birth weight to assess our confidence in our overall conclusion that there is an inverse relationship between PFOA and birth weight. First, we determined a hypothetical effect estimate necessary to shift our meta-analysis under two scenarios: *a*) that the 95% CI of the meta-analysis overlaps zero (becomes statistically insignificant), and *b*) that the meta-analysis effect estimate is greater than zero (moves in the opposite direction). We assumed that the new hypothetical study would have a standard error of 5.18 g/ng/mL, equal to the smallest in our group of studies (Fei et al. 2007). By inserting the values for the hypothetical study’s standard error and effect estimate into the meta-analysis, we found that another new study would have to have an effect estimate of 18 g/ng/mL in the positive direction in order to enlarge our CIs to overlap zero, and 225 g in the positive direction to shift our effect estimate to greater than zero. Second, to investigate how residual confounders might influence the meta-analysis, we conducted a separate meta-analysis using only unadjusted estimates from all the studies. Because Hamm et al. (2010) provided only an unadjusted estimate and *p*-value on a natural log (ln)-transformed scale, we made a log transformation for this study to obtain the untransformed estimate, and the standard error was calculated from the *p*-value (Altman and Bland 2011; Higgins et al. 2008). We found that the overall unadjusted estimate for change in birth weight was –30.9 (95% CI: –49.3, –12.5) g/ng/mL increase in serum or plasma PFOA. Compared with the effect estimate from the unadjusted meta-analysis, the adjusted estimate (–18.9 g) was closer to the null but more precise with less heterogeneity (unadjusted analysis: Cochran’s *Q* = 23.27, *p* = 0.002, *I*^2^ = 66%; adjusted analysis: Cochran’s *Q* = 12.92, *p* = 0.12, *I*^2^ = 38%).

*Quality of the body of evidence*. We did not downgrade or upgrade the rating of the human evidence on any of the criteria, resulting in an overall quality of the human evidence rating of “moderate” (Table 6). There were not enough studies to utilize a funnel plot analysis to assess publication bias. However, we did not find any suggestion of publication bias according to the considerations we assessed, that is, we conducted a comprehensive literature search and found studies of variable sizes and funding sources with generally consistent findings.

**Table 6:** Summary of findings, quality of evidence, and strength of evidence for PFOA and fetal growth.^*a*^

Quality factor^*b*^	Rating^*c*^	Basis
Downgrade
Risk of bias across studies	0	There is no indication that there is substantial risk of bias across the body of available evidence, particularly for the studies included in the meta-analysis.
Indirectness	0	The studies assessed population, exposure, and outcome of interest.
Inconsistency	0	With the exception of two small studies (Fromme et al. 2010; Kim S et al. 2011), results across studies are generally consistent in the magnitude and direction of effect estimates. The results of the meta-analysis for birth weight do not appear to be strongly influenced by an individual study. The results of all four meta-analyses for change in the fetal growth measures are consistent in the direction of overall effect estimates.
Imprecision	0	We judged that the CI of the meta-analysis for birth weight is sufficiently narrow.
Publication bias	0	We found no reason to suspect publication bias. The search was comprehensive, and the studies were generally consistent among their findings, regardless of size or funding source.
Upgrade
Large magnitude of effect	0	We did not consider the estimated effects large.
Dose response	0	Several studies in which association was modeled by categorized incremental exposure showed evidence of a dose–response relationship, but review authors agreed that the evidence was not compelling enough for an upgrade.
Confounding minimizes effect	0	We did not find evidence to suggest that possible residual confounders or biases would reduce effect estimate.
Overall quality of evidence (initial rating is ”moderate”)	Moderate	Moderate + (0) = moderate. (There were no upgrades or downgrades to change quality from the initial rating).
Summary of findings from meta-analysis	NA	We found decrements in fetal growth associated with PFOA exposure (see results from meta-analyses in Table 5).
Summary of qualitative findings	NA	Studies not included in the meta-analyses presented mixed results, mostly insignificant associations between PFOA and fetal growth (Figure 4; see also Supplemental Material, Figure S1).
Strength considerations
Quality of body of evidence	NA	Moderate
Direction of effect estimate	NA	Birth weight decreased with increasing exposure to PFOA.
Confidence in effect estimate	NA	It is unlikely that a new study would have an effect estimate that would make the results of the meta-analysis null or insignificant.
Other compelling attributes of the data that may influence certainty	NA	None
Overall strength of evidence	Sufficient	Based on our analysis and interpretation of the evidence, we concluded that there is a positive association between exposure and outcome, and we believe with reasonable confidence that chance, bias, and confounding can be ruled out as an explanation for the association. The available evidence includes results from one or more well-designed, well-conducted studies, and we believe that our conclusion is unlikely to be strongly affected by the results of future studies (see the definition in Table 3).
NA, not applicable. ^***a***^See the Supplemental Material of Lam et al. (2014) for additional details of rating quality and strength. ^***b***^Criteria for downgrading and upgrading quality are presented in Table 2. ^***c***^A “0” quality rating indicates there were no upgrades or downgrades for each factor being evaluated across the body of evidence.		

Strength of the body of evidence rating. Our strength of the evidence considerations were as follows:

Quality of body of evidence: moderate
Direction of effect estimate: decreasing birth weight with increasing exposure to PFOA
Confidence in effect estimate: unlikely that a new study would have an effect estimate that would make the results of the meta-analysis null or statistically insignificant
Other compelling attributes of the data that may influence certainty: none.

We compared these considerations to the definitions in Table 3 and concluded that there was “sufficient” human evidence that exposure to PFOA affects fetal growth in humans.

## Discussion

Based on this first application of the Navigation Guide systematic review methodology, we concluded that there was sufficient evidence of an association between PFOA exposure and reduced fetal growth. Our conclusion that the human data were sufficient was based on “moderate” quality evidence, a meta-analysis estimating a decrement in birth weight in relation to PFOA exposure in which we judged that the confidence bounds were narrow, and our confidence that a new study would be unlikely to have an effect estimate that would change the overall effect estimate of the meta-analysis. The smaller meta-analyses of other fetal growth measures were also consistent in the direction of the effect estimate.

The existence of unmeasured confounders will always be possible with observational studies, but we decided to not let this undermine our ability to make a statement about the available data. Additional information that may arise can and should inform future conclusions. We felt that we could rule out confounding “with reasonable confidence” (Table 3, definition of “Sufficient evidence of toxicity”) based on our assessment of the available data. We did not find any evidence suggesting substantial residual confounding. To get an idea of how residual confounding may influence the effect estimate of the association between PFOA exposure and birth weight, we conducted a meta-analysis using unadjusted estimates. Although the unadjusted meta-analysis had a larger effect estimate (i.e., adjusting for confounders attenuated the estimate), the CIs were wider and there was substantial heterogeneity among the unadjusted studies. As in the Bradford Hill considerations for causation, the GRADE approach considers consistency in effect estimates when evaluating confidence in the association and rating the quality of evidence (Schunemann et al. 2011). Because the effect estimates were more homogeneous after adjustment, we considered it more likely that the adjusted estimate was closer to the true association. If adjustment resulted in more heterogeneity, we would have been more concerned with potential residual confounding. Although this analysis does not prove that residual confounding does not exist, it did not uncover any evidence of unmeasured confounders, and we considered this as support for our interpretation that substantial effects of residual confounding are unlikely.

We also considered alternative hypotheses for the relationship between PFOA exposure and birth weight. For instance, an author of one of the studies included in our meta-analysis proposed that the pharmacokinetics of PFOA during pregnancy may influence the relationship between PFOA body burdens and fetal growth such that associations may be due to reverse causality (Whitworth et al. 2012). That is, mothers of lower-birth-weight babies might experience less plasma volume expansion and therefore reduced clearance of PFOA through glomerular filtration. To investigate the plausibility of an alternate hypothesis of reverse causation, we searched for evidence on the relationship between fetal growth and glomerular filtration rate, including relationships within the hypothesized causal pathway (i.e., between fetal growth and plasma volume expansion, and between plasma volume expansion and glomerular filtration rate) (for a list of studies that we systematically reviewed, see the Supplemental Material of Lam et al. 2014). Overall we found limited and inconsistent data that were inadequate to draw conclusions on the association between fetal growth and glomerular filtration rate. Thus, although we did not find evidence to suggest that the observed association between PFOA exposure and fetal growth can be explained, wholly or partially, by reverse causality, we cannot disprove this hypothesis. Nevertheless, we decided at this time there was no compelling evidence of reverse causation to justify altering our conclusions about the strength of the evidence. As others have pointed out (Savitz 2007), future studies should attempt to better separate biological determinants of body burdens and birth weight from a causal effect. In addition, experimental animal studies, in which dosing prior to outcome assessment precludes reverse causality, support our conclusions about the human data. On the basis of our companion review applying the Navigation Guide systematic review methodology to the nonhuman evidence, we concluded that there is sufficient evidence that fetal developmental exposure to PFOA reduces fetal growth in animals (Koustas et al. 2014).

We also considered that studies of the population that was highly exposed to PFOA through groundwater contamination found little evidence of an association with low birth weight (Nolan et al. 2009; Savitz et al. 2012a, 2012b; Stein et al. 2009) and on the continuous scale of birth weight (Savitz et al. 2012b). However, these studies differed from the studies included in our main meta-analysis with respect to exposure estimation as described in the risk of bias assessment; that is, these studies estimated exposure based on residence (ecological exposure), retrospective modeling of several parameters, or maternal postnatal exposure, and these studies primarily examined odds of low birth weight (< 2,500 g) rather than a change in birth weight on a continuous scale. We did not conduct a meta-analysis with odds ratios for low birth weight because so few studies (three populations) provided this measure and because a continuous change in birth weight provides more information than dichotomized birth weight. We did, however, conduct a meta-analysis including an effect estimate from one of the studies that retrospectively modeled exposure (Savitz et al. 2012b) and found minimal change in the results (Table 5); these results did not change our conclusions.

Although the magnitude of the effect estimate of PFOA on fetal growth may not be considered large at the individual or clinical level, it is important to consider implications at the population level. A relatively modest and subclinical effect size may be associated with substantial population burden if the exposure is prevalent (Bellinger 2012). From the meta-analysis we found an overall estimate of –18.9 (95% CI: –29.8, –7.9) g birth weight/ng/mL increase in serum or plasma PFOA (Figure 5). In the National Health and Nutrition Examination Survey 2003–2004, there was a 3.0-ng/mL difference in serum PFOA between the 50th and 95th percentiles of pregnant women (Woodruff et al. 2011b). This 3.0-ng/mL change, multiplied by the meta-analysis result of –18.9 g/ng/mL PFOA, yields a 56.7-g change in birth weight across these percentiles. To give a public health context to interpret this change in birth weight on a population level, we used 2010 U.S. National Vital Statistics birth weight data from the National Center for Health Statistics (NCHS) (Martin et al. 2012). The NCHS birth weight data are grouped into 500-g bins. We assumed that the data follow a skew *t*-distribution (Azzalini and Capitanio 2003), allowing us to fit a continuous distribution to the grouped data and producing a better fit to the data than assuming a normal distribution. Using the fitted distribution, the proportion of babies weighing < 2,500 g (low birth weight) was 8.6%. We estimated a 56.7-g increase in birth weight associated with a reduction in serum PFOA from the 95th to the 50th percentile. If the average birth weight in 2010 were increased by 56.7 g, the proportion of babies < 2,500 g would theoretically fall to 7.6% (95% CI: 7.0–8.2%), a reduction in the proportion of low birth weight babies in the U.S. of about 1%, or 40,000 babies in a year. However, because the population median of serum PFOA may be only about 3 ng/mL, this estimated benefit of reducing PFOA exposure and increasing birth weight may not be attributed equally across the population. Individuals with already low levels of PFOA (i.e., below the median) may not benefit, and individuals with the highest levels would benefit the most.

Our conclusion that there was sufficient evidence that developmental exposure to PFOA was associated with reduced fetal growth differed from the findings of an expert panel appointed to review the human health effects of PFOA (C8 Science Panel 2011). The panel concluded that PFOA was probably not linked to low birth weight and that the evidence of small reductions in average birth weight in relation to PFOA exposure was inconsistent. Our review occurred at a later date and therefore included more recent publications. These later publications (Chen et al. 2012; Maisonet et al. 2012; Whitworth et al. 2012) were included in our meta-analysis, showing consistent results and an overall reduction in birth weight associated with PFOA exposure. Our protocol specified contacting authors as a means to obtaining additional data or data on a scale that could be combined in a meta-analysis, and this contact proved essential in including many of the studies in the meta-analysis (Apelberg et al. 2007; Chen et al. 2012; Fromme et al. 2010; Kim S et al. 2011; Maisonet et al. 2012; Washino et al. 2009). In addition, by contacting authors of one of the included studies (Wang et al. 2011), we were alerted to an additional study on the same cohort under review at the time (Chen et al. 2012) and were able to include that study in the meta-analysis.

The meta-analysis provided a quantitative summary of the studies, combining and weighting studies to integrate information across multiple studies, and effectively increasing the power to detect an association among a group of studies that might otherwise appear to have disparate findings. Although there was a high level of consistency in the direction of the estimated effects except for one very small study in the meta-analysis (Kim S et al. 2011), a statistically significant inverse association between PFOA exposure and fetal growth was not detected in several individual studies, (Chen et al. 2012; Hamm et al. 2010; Washino et al. 2009; Whitworth et al. 2012).

The objective of our search was to be as comprehensive and inclusive of relevant research as possible. Our search identified 3,023 records, which were narrowed down to 17 during the title/abstract or full-text screening steps. Although our search retrieved many references that were irrelevant to our study question, because we applied prespecified exclusion criteria, screening the references was efficient. The average time to screen an abstract was 12 sec, and we excluded the majority of irrelevant references in < 10 hr. The process from search to rating the quality and strength of the human evidence was about 9 months.

A limitation to this review, and to all reviews in general, is that reviews are based on the available data, which may be insufficient in depth or breadth or may be otherwise limited. Future reviews could be strengthened if more investigators followed standardized reporting criteria such as the Strengthening the Reporting of Observational Studies in Epidemiology (STROBE) guidelines (von Elm et al. 2008), enabling improved quality assessment. In addition, we found that contacting study authors was essential to obtaining the data necessary to include some of the studies in the meta-analysis. Not all study authors were able to provide data that could be included in the meta-analysis. Future efforts in meta-analysis could also be supported by data repositories.

Our risk of bias tool also had limitations. Although there is existing guidance for assessing risk of bias of human observational studies (Viswanathan et al. 2012; Wells 2014), there is no universally accepted tool (Sanderson et al. 2007). The risk of bias domains “exposure assessment” and “confounding” were less developed than other domains that were transferred more directly from established evidence-based risk of bias tools. Additionally, in future reviews, we will consider the assessment of outcome as a separate risk of bias domain. For this case study, potential bias resulting from outcome misclassification fell under “other” risk of bias and was not a problematic risk because the outcomes were standard birth measurements that did not vary across study groups. However, it is possible that in future cases of other outcomes more attention will need to be given to potential bias in the assessment of those outcomes.

Because we were simultaneously developing and applying the Navigation Guide method, a limitation of this review is that we did not anticipate and define *a priori* all the benchmarks we ultimately used for rating the quality and the strength of the evidence, such as our analysis of what a new study would have to find in order to change our confidence in the effect estimate and direction of the meta-analysis. In assessing quality and strength according to factors and considerations that had not been prespecified, we conducted further analysis and abided by GRADE’s principle to be conservative in changing the rating of the body of evidence up or down (Balshem et al. 2011). It may be impossible to anticipate all instances for which a judgment or decision must be made in the conduct of a systematic review; therefore, the principles we used for addressing these instances will be integrated into future protocols. A protocol, a set of instructions, and definitions does not, however, take the place of expert judgment. The strength of systematic review methods is that, as new studies become available, a conclusion can be systematically and transparently reevaluated. Finally, the components of the Navigation Guide methodology that were not taken from empirically supported preexisting methods need validation in future cases.

## Conclusion

On the basis of our evaluation and the Navigation Guide criteria, we concluded that there is sufficient evidence of an association between PFOA exposure and reduced fetal growth. There may be remaining uncertainty. However, we investigated residual confounding and evidence for reverse causality via reduced renal clearance, and despite the cross-sectional nature of the human evidence, our judgment was that chance, bias, and confounding could be ruled out with reasonable confidence. The proof-of-concept case study demonstrates the use of the Navigation Guide to efficiently apply the rigor and transparency of systematic review methodology to environmental health questions. The method does not take the place of expert judgment, but it requires transparency in the rationale exercised by the experts. Further refinement and proof-of-concept applications of the Navigation Guide methodology will continue, with the ultimate goal of supporting timely evidence-based recommendations for the prevention of harm to public health.

## Supplemental Material

(1.4 MB) PDF
