# Supplementary material for: The Navigation Guide—Evidence-Based Medicine Meets Environmental Health: Systematic Review of Human Evidence for PFOA Effects on Fetal Growth
Source: Environ Health Perspect. 2014 Jun 25;122(10):1028–39. doi: 10.1289/ehp.1307893 (PMC4181929; doi:10.1289/ehp.1307893)
Supplement: (1.4 MB) PDF [file ehp.1307893.s001.508.pdf]

Environmental Health Perspectives doi:10.1289/ehp.1307893

Supplemental Material

The Navigation Guide—Evidence-Based Medicine Meets Environmental Health: Systematic Review of Human Evidence for PFOA Effects on Fetal Growth

Paula I. Johnson, Patrice Sutton, Dylan S. Atchley, Erica Koustas, Juleen Lam, Saunak Sen, Karen A. Robinson, Daniel A. Axelrad, and Tracey J. Woodruff

Table of Contents **Page**

Table S1. Search terms used in systematic literature search. 3

Table S2. Search results from systematic literature search (main databases using search terms in Table S1 and additional websites). 5

Table S3. Characteristics of Apelberg et al. 2007. 7

Table S4. Characteristics of Arbuckle et al. 2012. 8

Table S5. Characteristics of Chen et al. 2012. 9

Table S6. Characteristics of Fei et al. 2008. 10

Table S7. Characteristics of Fei et al. 2007. 11

Table S8. Characteristics of Fromme et al. 2010. 12

Table S9. Characteristics of Halldorsson et al. 2012. 13

Table S10. Characteristics of Hamm et al. 2010. 14

Table S11. Characteristics of Kim S et al. 2011. 15

Table S12. Characteristics of Kim SK et al. 2011. 16

Table S13. Characteristics of Maisonet et al. 2012. 17

Table S14. Characteristics of Monroy et al. 2008. 19

Table S15. Characteristics of Nolan et al. 2009. 20

Table S16. Characteristics of Savitz et al. 2012a. 21

Table S17. Characteristics of Savitz et al. 2012b (Study 1). 22

Table S18. Characteristics of Savitz et al. 2012b (Study 2). 23

Table S19. Characteristics of Stein et al. 2009. 25

Table S20. Characteristics of Washino et al. 2009. 26

Table S21. Characteristics of Whitworth et al. 2012. 27

Table S22. Meta-analysis for association between PFOA exposure and birth length (cm). 28

Table S23. Meta-analysis for association between PFOA exposure and ponderal index. 29

Table S24. Meta-analysis for association between PFOA exposure and head circumference (cm). 30

References 31

Instructions for Making Risk of Bias Determinations 33

Figure S1. Summary of data extracted from all studies of PFOA exposure with a dichotomous outcome of birth weight. 43

Table S1. Search terms used in systematic literature search.

Search	PubMed
#1 Substance terms	335-67-1 [rn] OR perfluorooctanoic acid [nm] OR (perfluorooctanoic acid [tiab] OR perfluorooctanoic acids [tiab]) OR (perfluoroctanoic acid [tiab] OR perfluoroctanoic acids [tiab]) OR (perfluoro-n-octanoic acid [tiab] OR perfluoro-n-octanoic acids [tiab]) OR (pentadecafluorooctanoic acid [tiab] OR pentadecafluorooctanoic acids [tiab]) OR APFO [tiab] OR (perfluorinated [tiab] AND octanoic acid [tiab]) OR (perfluorinated [tiab] AND octanoic acids [tiab]) OR (perfluorooctanoate [tiab] OR perfluorooctanoates [tiab]) OR perfluorooctanoyl chloride [tiab] OR PFOA [tiab] OR (fluorinated telomer alcohol [tiab] OR fluorinated telomer alcohols [tiab]) OR (fluoro-telomer alcohol [tiab] OR fluoro-telomer alcohols [tiab]) OR (fluorocarbon emulsion [tiab] OR fluorocarbon emulsions [tiab]) OR (perfluorocarbon [tiab] OR perfluorocarbons [tiab]) OR (fluorocarbon polymer [tiab] OR fluorocarbon polymers [tiab]) OR (fluorinated polymer [tiab] OR fluorinated polymers [tiab]) OR octanoic acids [mh] OR (octanoic acid [tiab] OR octanoic acids [tiab]) OR caprylates [mh] OR (caprylate [tiab] OR caprylates [tiab]) OR (polyfluoroalkyl [tiab] OR polyfluoroalkyls [tiab] OR polyfluoroalkylated [tiab]) OR PFAA [tiab] OR (perfluoroalkyl chemical [tiab] OR perfluoroalkyl chemicals [tiab]) OR (c8 [tiab] AND perfluorinated [tiab]) OR (fluoropolymer [tiab] OR fluoropolymers [tiab] OR fluoropolymeric [tiab]) OR (fluorosurfactant [tiab] OR fluorosurfactants [tiab]) OR (perfluorochemical [tiab] OR perfluorochemicals [tiab]) OR PFCs [tiab] OR (perfluoroalkyl carboxylate [tiab] OR perfluoroalkyl carboxylates [tiab]) OR (perfluorocarboxylate [tiab] OR perfluorocarboxylates [tiab]) OR PFCA [tiab] OR (perfluorinated carboxylic acid [tiab] OR perfluorinated carboxylic acids [tiab]) OR FC 143 [tiab] OR (pentadecafluorooctanoate [tiab] OR pentadecafluorooctanoates [tiab])
#2 Reproductive/developmental toxicity terms	developmental biology [mh] OR developmental biology [tiab] OR embryonic and fetal development [mh] OR (embryonic [tiab] OR embryonically [tiab]) OR fetal development [tiab] OR growth and development [mh] OR growth and development [subheading] OR development [tiab] OR developmental [tiab] OR developmentally [tiab] OR embryology [mh] OR embryology [tiab] OR ecotoxicology [mh] OR ecotoxicology [tiab] OR ecology [mh] OR ecology [tiab] OR ecological [tiab] OR ecologically [tiab] OR toxicology [mh] OR (toxicology [tiab] OR toxicological [tiab] OR toxicologically [tiab]) OR toxicogenetics [mh] OR (toxicogenetic [tiab] OR toxicogenetics [tiab]) OR growth [mh] OR growth [tiab] OR environment and public health [mh] OR environment and public health [tiab] OR body weight [mh] OR (weight [tiab] OR weights [tiab] OR weighed [tiab]) OR birthweight [tiab] OR infant, low birth weight [mh] OR embryo loss [mh] OR (embryo loss [tiab] OR embryo losses [tiab]) OR gestational age [mh] OR (gestational age [tiab] OR gestational ages [tiab]) OR endocrine disruptors [mh] OR (endocrine disruptor [tiab] OR endocrine disruptors [tiab] OR endocrine disruption [tiab]) OR reproduction [mh] OR reproduction [tiab] OR toxicity [subheading] OR toxicity [tiab] OR (toxic [tiab] OR toxics [tiab])
#3 Human study terms	**#1 AND #2 NOT (animals[mh] NOT humans[mh])**

Search	PubMed
#1 Substance terms	'perfluorooctanoic acid' OR 'perfluoro n octanoic acid' OR 'pentadecafluorooctanoic acid' OR apfo OR 'perfluorinated octanoic acid' OR perfluorooctanoate OR 'perfluorooctanoyl chloride' OR pfoa OR 'fluorinated telomer alcohol' OR 'fluorinated telomer alcohols' OR 'fluoro telomer alcohol' OR 'fluoro telomer alcohols' OR 'fluorocarbon emulsion' OR perfluorocarbon* OR 'fluorocarbon polymer' OR 'fluorocarbon polymers' OR 'fluorinated polymer' OR 'fluorinated polymers' OR polyfluoroalkyl* OR pfaa OR 'perfluoroalkyl chemical' OR 'perfluoroalkyl chemicals' OR c8 OR perfluorochemical* OR pfcs OR 'perfluoroalkyl carboxylate' OR perfluorocarboxylate OR pfca OR 'perfluorinated carboxylic acid' OR pentadecafluorooctanoate
#2 Reproductive/developmental toxicity terms	'developmental biology' OR 'embryonic fetal development' OR embryonic* OR 'fetal development' OR 'growth and development' OR development* OR embryology OR ecotoxicology OR ecolog* OR toxic* OR toxicol* OR toxicogenetic* OR growth OR 'environment and public health' OR 'body weight' OR 'body weights' OR 'birth weight' OR 'birth weights' OR birthweight* OR 'infant, low birth weight' OR 'embryo loss' OR 'embryo losses' OR 'gestational age' OR 'gestational ages' OR 'endocrine disruption' OR 'endocrine disrupting' OR reproduction
#3 Human study terms	epidemio* OR cohort OR participant* OR questionnaire
**#4**	**#1 AND #2 AND #3**
#1 Substance terms	TS=(perfluorooctanoic acid* OR perfluoro-n-octanoic acid* OR pentadecafluorooctanoic acid* OR APFO OR perfluorinated and octanoic acid* OR perfluorooctanoate* OR perfluorooctanoyl chloride OR PFOA OR fluorinated telomer alcohol* OR fluoro-telomer alcohol* OR fluorocarbon emulsion* OR perfluorocarbon* OR fluorocarbon polymer* OR fluorinated polymer* OR octanoic acid* OR caprylate* OR polyfluoroalkyl* OR PFAA OR perfluoroalkyl chemical* OR c8 and perfluorinated OR fluoropolymer* OR fluorosurfactant* OR perfluorochemical* OR PFCs OR perfluoroalkyl carboxylate* OR perfluorocarboxylate* OR PFCA OR perfluorinated carboxylic acid* OR pentadecafluorooctanoate*)
#2 Reproductive/developmental toxicity terms	TS=(developmental biology OR embryonic and fetal development OR embryonic* OR fetal development OR growth and development OR development* OR embryology OR ecotoxicology OR ecolog* OR toxic* OR toxicol* OR toxicogenetic* OR growth OR environment and public health OR body weight* OR birth weight* OR birthweight* OR infant, low birth weight OR embryo loss* OR gestational age* OR endocrine disrupt* OR reproduction)
#3 Human study terms	(TS=epidemio* OR TS=cohort OR TS=population* OR TS=participant* OR TS=exposure* OR TS= questionnaire OR SO=epidemio*)
**#4**	**#1 AND #2 AND #3 NOT SO=polymer ***

Table S2. Search results from systematic literature search (main databases using search terms in Table S1 and additional websites).

**Source**[a]	**No. records retrieved**[b]
PubMed	2,268
Embase	457
Web of Science	545
ATSDR Toxicological Profiles http://www.atsdr.cdc.gov/toxpro2.html	1
CalEPA Office of Environmental Health Hazard Assessment http://www.oehha.ca.gov/risk.html	135
Chem ID http://chem.sis.nlm.nih.gov/chemidplus/	1
Chemfinder http://www.chemspider.com	8
Chemical Carcinogenesis Research Information System http://www.nlm.nih.gov/pubs/factsheets/ccrisfs.html	2
DART http://toxnet.nlm.nih.gov/cgi-bin/sis/htmlgen?DARTETIC	11
EPA IRIS internet http://www.epa.gov/iris	3
EPA NSCEP http://www.epa.gov/ncepihom/	301
EPA Science Inventory http://cfpub.epa.gov/si/index.cfm	344
EPA Substance Registry System http://www.epa.gov/srs/	8
European Chemicals Agency http://echa.europa.eu/home_en.asp	6
Hazardous Substances Data Bank http://toxnet.nlm.nih.gov/cgi-bin/sis/htmlgen?HSDB	2
IARC http://monographs.iarc.fr/htdig/search.html	2
IPCS INCHEM http://www.inchem.org/	1
ITER http://iter.ctcnet.net/publicurl/pub_search_list.cfm	1
NIOSHTIC 2 http://www2.cdc.gov/nioshtic 2/Nioshtic2.htm	13
US National Toxicology Program http://ntpserver.niehs.nih.gov/main_pages/NTP_ALL_STDY_PG.html	59
TERA http://www.tera.org/	18
WHO assessments http://www.who.int/ipcs/assessment/en/	17
Toxline http://toxnet.nlm.nih.gov/cgi-bin/sis/htmlgen?TOXLINE	166
USEPA Health and Environmental Studies Online http://hero.epa.gov	127
TSCA Test Submissions http://www.ntis.gov/products/ots.aspx	136
EPA Docket http://www.regulations.gov/	41

[a]Table presents databases for which search results were returned; databases that did not return search results follow: ATSDR Interaction Profiles http://www.atsdr.cdc.gov/interactionprofiles/; EPA Acute Exposure Guideline Levels

http://www.epa.gov/oppt/aegl/chemlist.htm; EPA IRIS edocket and official records; GENETOX http://toxnet.nlm.nih.gov/cgi-bin/sis/htmlgen?GENETOX; Health Canada First Priority List Assessments http://www.hcsc.gc.ca/hecs sesc/exsd/psl1.htm; Health Canada Second Priority List Assessments http://www.hcsc.gc.ca/hecs sesc/exsd/psl2.htm; ILSI http://www.ilsi.org/. [b]PubMed, Web of Science and Embase searches were performed on April 30, May 7 and May 11, 2012, respectively; all remaining database searches were performed April 23-27, 2012.

Table S3. Characteristics of Apelberg et al. 2007.

Bias	Rating	Support for judgment
Recruitment	Low risk	The strategy for recruiting participants was consistent across study groups.
Blinding	Low risk	Knowledge of the exposure groups was adequately prevented during the study.
Confounding	Low risk	The authors adjusted for both maternal age and gestational age in their analysis.
Exposure assessment	Low risk	The exposure assessment methods were robust and included a detailed description of QA/QC.
Incomplete outcome data	Low risk	The study did not have incomplete outcome data.
Selective reporting	Low risk	The study is free of suggestion of selective outcome reporting. All of the study's specified outcomes were adequately reported.
Other bias	Probably low risk	Authors were unable to collect cord blood for 42 of 341 participants because infants were lower gestational age and birth weight; and therefore, were not included in analysis.
Conflict of interest	Low risk	The authors report no conflict of interest, and associated funds and persons appear to be from government and/or academia only.

- Design: Cross-sectional study
- Participants: Singleton deliveries in Baltimore, MD between November 2004 and March 2005 (Baltimore THREE study).
- Exposure: PFOA concentrations in cord blood (continuous). n=293.
- Outcomes: Birth weight (continuous; beta statistic), head circumference, length, ponderal index.
- Notes: Received beta estimates on untransformed PFOA scale from study author (Goldman 2012).

Table S4. Characteristics of Arbuckle et al. 2012.

Bias	Rating	Support for judgment
Recruitment	Probably low risk	The study lacks description of recruitment methods such as number of individuals approached, but otherwise no reason to suspect there were substantial differences between comparison groups other than PFOA exposure.
Blinding	Probably low risk	The authors did not discuss blinding but the study design prevents knowledge of exposure groups.
Confounding	High risk	The authors did not adjust for gestational age or maternal age in their analysis.
Exposure assessment	Low risk	The exposure assessment methods were robust and included a detailed description of QA/QC.
Incomplete outcome data	Low risk	Incomplete outcome data were adequately addressed.
Selective reporting	Low risk	The study is free of suggestion of selective outcome reporting. All of the study's specified outcomes were adequately reported.
Other bias	Low risk	No other potential biases are suspected.
Conflict of interest	Probably low risk	Associated funds and persons appear to be from government and/or academia only and free of financial interests in study results. However, no claim denying conflicts of interest was made.

- Design: Cross-sectional study
- Participants: Women at Ottawa Hospital during 2005-2008. Majority were scheduled for cesarean schedule.
- Exposure: PFOA concentrations in cord blood (continuous). n=126.
- Outcomes: Birth weight (dichotomous; beta statistic).
- Notes: Birth weight was a covariate in model of ln(PFOA)

Table S5. Characteristics of Chen et al. 2012.

Bias	Rating	Support for judgment
Recruitment	Low risk	The strategy for recruiting participants was consistent across study groups.
Blinding	Low risk	Knowledge of the exposure groups was adequately prevented during the study.
Confounding	Low risk	The authors accounted for both maternal age and gestational age in their analysis.
Exposure assessment	Low risk	The exposure assessment methods were robust and included a detailed description of QA/QC.
Incomplete outcome data	Low risk	Incomplete outcome data were adequately addressed.
Selective reporting	Low risk	The study is free of suggestion of selective outcome reporting. All of the study's specified outcomes were adequately reported.
Other bias	Low risk	No other potential biases are suspected.
Conflict of interest	Low risk	The authors report no conflict of interest, and associated funds and persons appear to be from government and/or academia only.

- Design: Cross-sectional study.
- Participants: Non-smoking mothers in Taipei between April 2004 and January 2005. The subjects were from the Taiwan Birth Panel Study.
- Exposure: PFOA concentrations in cord blood (continuous). n=429.
- Outcomes: Birth weight (continuous; beta statistic, odds ratio), birth length, head circumference, ponderal index, small for gestational age.
- Notes: Study was included after contacting authors of a paper that was identified during the systematic search (Wang et al. 2011). The two papers are written on the same cohort, but the Chen et al paper has fetal growth measurements as outcomes of the analyses (under peer review at time of search).

Table S6. Characteristics of Fei et al. 2008.

Bias	Rating	Support for judgment
Recruitment	Probably low risk	Study lacks complete description of recruitment methods, but otherwise no reason to suspect there were substantial differences between comparison groups other than PFOA exposure.
Blinding	Low risk	Knowledge of the exposure groups were adequately prevented during the study.
Confounding	Low risk	The authors adjusted for both maternal age and gestational age in their analysis.
Exposure assessment	Probably low risk	Incomplete description of the quality assurance and control aspects of the exposure measurements as outlined in the protocol, but otherwise no problems relating to exposure misclassification were detected.
Incomplete outcome data	Low risk	Incomplete outcome data were adequately addressed.
Selective reporting	Low risk	The study is free of suggestion of selective outcome reporting. All of the study's specified outcomes were adequately reported.
Other bias	Low risk	No other potential biases are suspected.
Conflict of interest	High risk	3M, a manufacturer of PFOA, provided funding for the study and conducted laboratory analysis

- Design: Cross-sectional study.
- Participants: Randomly selected group of singleton births from Danish National Birth Cohort between 1996 and 2002.
- Exposure: PFOA concentrations in maternal plasma collected in the 1st trimester (Continuous and quartile). n=1399.
- Outcomes: Birth length, head circumference, abdominal circumference.
- Notes: Ponderal index was also analyzed but only presented as stratified by certain covariates.

Table S7. Characteristics of Fei et al. 2007.

Bias	Rating	Support for judgment
Recruitment	Probably low risk	Study lacks description of recruitment methods, but otherwise no reason to suspect there were substantial differences between comparison groups other than PFOA exposure.
Blinding	Low risk	Knowledge of the exposure groups were adequately prevented during the study.
Confounding	Low risk	The authors adjusted for both maternal age and gestational age in their analysis.
Exposure assessment	Probably low risk	Incomplete description of the quality assurance and control aspects of the exposure measurements as outlined in the protocol, but otherwise no problems relating to exposure misclassification were detected.
Incomplete outcome data	Low risk	Incomplete outcome data were adequately addressed.
Selective reporting	Low risk	The study is free of suggestion of selective outcome reporting. All of the study's specified outcomes were adequately reported.
Other bias	Low risk	No other potential biases are suspected.
Conflict of interest	High risk	3M, a manufacturer of PFOA, provided funding for the study and conducted laboratory analysis.

- Design: Cross-sectional study.
- Participants: Randomly selected group of singleton births from Danish National Birth Cohort between March 1996 and November 2002.
- Exposure: PFOA concentrations in maternal plasma collected in the 1st trimester (Continuous and quartile). n=1387.
- Outcomes: Birth weight (continuous and dichotomous; beta statistic, difference in means, odds ratio).

Table S8. Characteristics of Fromme et al. 2010.

**Bias**	**Rating**	**Support for judgment**
Recruitment	Low risk	The strategy for recruiting participants was consistent across study groups.
Blinding	Probably low risk	The authors did not discuss blinding but the study design prevents knowledge of exposure groups.
Confounding	High risk	Gestational age was not measured in the study.
Exposure assessment	Low risk	The exposure assessment methods were robust and included a detailed description of QA/QC.
Incomplete outcome data	Low risk	The number participants reported in the paper is larger than the n provided as raw data, but the discrepancy was explained in correspondence with the author.
Selective reporting	Low risk	The study is free of suggestion of selective outcome reporting. All of the study's specified outcomes were adequately reported.
Other bias	Low risk	No other potential biases are suspected.
Conflict of interest	Probably low risk	Associated funds and persons appear to be from government and/or academia only and free of financial interests in study results. However, no claim denying conflicts of interest was made.

- Design: Cross-sectional study.
- Participants: Mothers in Munich, Germany participating in a birthing class between December 2007 and October 2009.
- Exposure: PFOA concentrations in maternal serum drawn between 24-37 weeks gestation (n = 44) and at delivery (n = 38). Also, PFOA concentrations in cord blood at delivery (n = 33).
- Outcomes: none.
- Notes: Received raw individual data on birth weight and PFOA concentrations from study authors.

Table S9. Characteristics of Halldorsson et al. 2012.

Bias	Rating	Support for judgment
Recruitment	Low risk	The strategy for recruiting participants was consistent across study groups.
Blinding	Probably low risk	The authors did not discuss blinding but the study design prevents knowledge of exposure groups.
Confounding	High risk	The authors did not adjust for gestational age or maternal age in their analysis.
Exposure assessment	Low risk	The exposure assessment methods were robust and included a detailed description of QA/QC.
Incomplete outcome data	Low risk	The study did not have incomplete outcome data.
Selective reporting	Low risk	The study is free of suggestion of selective outcome reporting. All of the study's specified outcomes were adequately reported.
Other bias	Low risk	No other potential biases are suspected.
Conflict of interest	Low risk	The authors report no conflict of interest, and associated funds and persons appear to be from government and/or academia only.

- Design: Cross-sectional study.
- Participants: Singleton pregnancies in Aarhus, Denmark between April 1988 and January 1989.
- Exposure: PFOA concentrations in maternal serum drawn at 30 weeks gestation. n = 665.
- Outcomes: Waist circumference and BMI of offspring at age 20.
- Notes: Birth weight was presented by quartiles of maternal PFOA concentration in a table of maternal characteristics.

Table S10. Characteristics of Hamm et al. 2010.

Bias	Rating	Support for judgment
Recruitment	Low risk	The strategy for recruiting participants was consistent across study groups.
Blinding	Probably low risk	The authors did not discuss blinding but the study design prevents knowledge of exposure groups.
Confounding	Low risk	The authors adjusted for both maternal age and gestational age in their analysis.
Exposure assessment	Low risk	The exposure assessment methods were robust and included a detailed description of QA/QC.
Incomplete outcome data	Low risk	The study did not have incomplete outcome data.
Selective reporting	Low risk	The study is free of suggestion of selective outcome reporting. All of the study's specified outcomes were adequately reported.
Other bias	Probably low risk	Because the study population was selected from subjects undergoing a screening procedure typically recommended for older women, the study could be biased towards inclusion of older women and high-risk pregnancies. However, no association between PFOA and age was found, and there was no reason to suspect that comparison groups within the study were different with respect to PFOA exposure.
Conflict of interest	Low risk	The authors report no conflict of interest, and associated funds and persons appear to be from government and/or academia only.

- Design: Cross-sectional study.
- Participants: Singleton births from women (>18 years) that elected to undergo second trimester prenatal "triple-screen" between 12/15/05 and 6/22/06 in Edmonton, Canada.
- Exposure: PFOA concentrations in maternal serum collected at 15-16 weeks of gestation (Continuous and tertile). n=252.
- Outcomes: Birth weight (continuous; beta statistic, z-score), small for gestational age.

Table S11. Characteristics of Kim S et al. 2011.

Bias	Rating	Support for judgment
Recruitment	Probably low risk	Study lacks complete description of exclusion criteria, but otherwise no reason to suspect there were substantial differences between comparison groups other than PFOA exposure.
Blinding	Low risk	Knowledge of the exposure groups were adequately prevented during the study.
Confounding	Low risk	The authors adjusted for both maternal age and gestational age in their analysis.
Exposure assessment	Low risk	The exposure assessment methods were robust and included a detailed description of QA/QC.
Incomplete outcome data	Low risk	There was a discrepancy in the sample sizes for data analysis from what was reported as the total number of participants. However, the discrepancy was explained as participant drop-out and missing covariate information upon correspondence with the authors.
Selective reporting	Low risk	The study is free of suggestion of selective outcome reporting. All of the study's specified outcomes were adequately reported.
Other bias	Low risk	No other potential biases are suspected.
Conflict of interest	Probably low risk	Associated funds and persons appear to be from government and/or academia only and free of financial interests in study results. However, no claim denying conflicts of interest was made.

- Design: Cross-sectional study.
- Participants: Pregnant women at three hospitals in Seoul, Cheongju, and Gumi, South Korea between August 2008 and March, 2009.
- Exposure: PFOA concentrations in maternal serum collected in the 3rd trimester and cord blood (continuous). n=31 (maternal serum); n=43 (cord serum).
- Outcomes: Birth weight (continuous; Pearson's partial correlation).
- Notes: Received raw individual level data from authors.

Table S12. Characteristics of Kim SK et al. 2011.

**Bias**	**Rating**	**Support for judgment**
Recruitment	Probably low risk	Study lacks complete description of recruitment criteria, but otherwise no reason to suspect there were substantial differences between comparison groups other than PFOA exposure.
Blinding	Probably low risk	The authors did not discuss blinding but the study design prevents knowledge of exposure groups.
Confounding	High risk	The authors did not adjust for gestational age in their analysis.
Exposure assessment	Low risk	The exposure assessment methods were robust and included a detailed description of QA/QC.
Incomplete outcome data	Low risk	Three participants were excluded from the birth weight analysis, but the discrepancy was accounted for in correspondence with the authors.
Selective reporting	Low risk	The study is free of suggestion of selective outcome reporting. All of the study's specified outcomes were adequately reported.
Other bias	Low risk	No other potential biases are suspected.
Conflict of interest	High risk	No conflict of interest statement is provided and the study was funded by a plastic additives manufacturer.

- Design: Cross-sectional study.
- Participants: Women (age >25) in Seoul Korea in 2007.
- Exposure: PFOA concentrations in maternal serum collected the day before delivery and cord blood (continuous). n=17.
- Outcomes: Birth weight (continuous; spearman correlation p-value).

Table S13. Characteristics of Maisonet et al. 2012.

Bias	Rating	Support for judgment
Recruitment	Probably low risk	The population is divided into two groups (menarche <11.5 yrs. and random non-cases >11.5 yrs.) from previous study and randomization is not described. Weighted statistical analysis accounted for sampling scheme.
Blinding	Probably low risk	The authors did not discuss blinding but the study design prevents knowledge of exposure groups.
Confounding	Low risk	The authors only adjusted for gestational age in their analysis because preliminary analysis found that maternal age did not have an effect in this population.
Exposure assessment	Low risk	The exposure assessment methods were robust and included a detailed description of QA/QC.
Incomplete outcome data	Probably low risk*	A number of births was excluded from analysis due to missing covariate data (6% for birth weight, 20% for birth length). Categories for variables missing >10% rate were included in analyses. No reason to suspect that study groups are different with respect to missing covariates; mean values of outcomes and covariates were similar between group in analysis and total cohort.
Selective reporting	Low risk	The study is free of suggestion of selective outcome reporting. All of the study's specified outcomes were adequately reported.
Other bias	Low risk	No other potential biases are suspected.
Conflict of interest	Low risk	The authors report no conflict of interest, and associated funds and persons appear to be from government and/or academia only.

- Design: Prospective cohort study; cross-sectional exposure.
- Participants: Pregnant women from the Avon Longitudinal Study for Parents and Children, Great Britain with an expected delivery data between April, 1991 and December, 1992 selected from a previous study of maternal perfluoroalkyl compound concentrations and menarche.
- Exposure: PFOA concentrations in maternal serum collected between 10-28 weeks of gestation (categorical and tertile). n=439.
- Outcomes: Birth weight (continuous; beta statistic), length, ponderal index.

- Notes: Cross-sectional exposure. Received beta estimates on untransformed PFOA scale from authors. *Because the level of missing data was higher (19-20%) for birth length and ponderal index, we assigned “probably high risk” to these outcomes (not presented in Figure 3) for this domain. This is the only instance where a risk of bias rating differed by outcome and therefore risk of bias results are not presented separately by outcome.

Table S14. Characteristics of Monroy et al. 2008.

Bias	Rating	Support for judgment
Recruitment	Low risk	The strategy for recruiting participants was consistent across study groups.
Blinding	Probably low risk	The authors did not discuss blinding but the study design prevents knowledge of exposure groups.
Confounding	High risk	The authors did not adjust for maternal age in their analysis.
Exposure assessment	Low risk	The exposure assessment methods were robust and included a detailed description of QA/QC.
Incomplete outcome data	Low risk	Incomplete outcome data were adequately addressed.
Selective reporting	Low risk	The study is free of suggestion of selective outcome reporting. All of the study's specified outcomes were adequately reported.
Other bias	Low risk	No other potential biases are suspected.
Conflict of interest	High risk	The study was funded by the American Chemistry Council, an industry group.

- Design: Cross-sectional study.
- Participants: Women that presented for an obstetrical ultrasound from January 2004 until June 2005 in Ontario Canada. The subjects are part of a larger ongoing cohort study, Family Study.
- Exposure: PFOA concentrations in maternal serum collected in the 2nd trimester and cord serum (continuous). n=89.
- Outcomes: Birth weight (continuous; beta statistic).
- Notes: Birth weight was modeled as a predictor of PFOA levels.

Table S15. Characteristics of Nolan et al. 2009.

**Bias**	**Rating**	**Support for judgment**
Recruitment	Low risk	The strategy for recruiting participants was consistent across study groups.
Blinding	Probably low risk	The authors did not discuss blinding but the study design prevents knowledge of exposure groups.
Confounding	Low risk	The authors adjusted for both maternal age and gestational age in their analysis.
Exposure assessment	High risk	Exposure was estimated using water service category and participant resident history.
Incomplete outcome data	Low risk	The study did not have incomplete outcome data.
Selective reporting	Low risk	The study is free of suggestion of selective outcome reporting. All of the study's specified outcomes were adequately reported.
Other bias	Low risk	No other potential biases are suspected.
Conflict of interest	Low risk	The authors report no conflict of interest, and associated funds and persons appear to be from government and/or academia only.

- Design: Cross-sectional study.
- Participants: Singleton births between January 1, 2003 and September 1, 2005 in Washington County, Ohio.
- Exposure: Estimated PFOA exposure based on water service category (water provided completely, partially, or not at all by PFOA-contaminated source). n=1555.
- Outcomes: Birth weight (continuous and dichotomous; difference in means, beta statistic, odds ratio).

Table S16. Characteristics of Savitz et al. 2012a.

Bias	Rating	Support for judgment
Recruitment	Low risk	The strategy for recruiting participants was consistent across study groups.
Blinding	Probably high risk	The outcome data were self-reported and it was unclear, although suspected, that the participants were aware of being exposed to PFOA because they were part of a publicized study that was funded by a lawsuit.
Confounding	Probably high risk	The authors restricted the analysis of birth weight to term births but did not account for gestational age in the analysis of term birth weight.
Exposure assessment	Probably high risk	Exposure was retrospectively estimated using environmental, exposure and pharmacokinetic modeling with several assumptions, although moderate correlation between predicted vs. observed serum PFOA was reported. Modeling used self-reported residential histories and GIS linkage to public water distribution systems.
Incomplete outcome data	High risk	Unclear why number of term and preterm births does not add up to number of live births reported; appears that a subset (n=120) of births was not accounted for in analysis of low birth weight.
Selective reporting	Low risk	The study is free of suggestion of selective outcome reporting. All of the study's specified outcomes were adequately reported.
Other bias	Low risk	No other potential biases are suspected.
Conflict of interest	Low risk	The authors report no conflict of interest, and associated funds and persons appear to be from government and/or academia only.

- Design: Retrospective cohort study.
- Participants: Singleton pregnancies between 1990 and 2006 in the Mid-Ohio Valley. Participants are part of the much larger C8 Health Project. n=10,189 total live births.
- Exposure: PFOA exposure estimated retrospectively based on environmental, exposure and pharmacokinetic modeling in conjunction with self-reported residential histories and GIS linkage to public water distribution systems. The correlation between the predicted and observed serum PFOA (measured at time of C8 Health Project) was r=0.67. PFOA concentrations were assigned to calendar year of pregnancy for present study data analysis (continuous and quintiles of exposure used in analysis).
- Outcomes: Birth weight (dichotomous; odds ratio).
- Notes: Although excluded from analysis of PFOA and term low birth weight, the relationship between PFOA and pre-term birth was examined separately (findings: non-significant adjusted OR).

Table S17. Characteristics of Savitz et al. 2012b (Study 1).

Bias	Rating	Support for judgment
Recruitment	Low risk	The strategy for recruiting participants was consistent across study groups.
Blinding	Probably low risk	The authors did not discuss blinding but the study design prevents knowledge of exposure groups.
Confounding	Low risk	The authors adjusted for both maternal age and gestational age in their analysis.
Exposure assessment	High risk	Exposure was retrospectively estimated using environmental, exposure and pharmacokinetic modeling with several assumptions, although moderate correlation between predicted vs. observed serum PFOA was reported. Modeling used birth residence for 6 yr. residential history.
Incomplete outcome data	Low risk	The study did not have incomplete outcome data.
Selective reporting	Low risk	The study is free of suggestion of selective outcome reporting. All of the study's specified outcomes were adequately reported.
Other bias	Low risk	No other potential biases are suspected.
Conflict of interest	Low risk	The authors report no conflict of interest, and associated funds and persons appear to be from government and/or academia only.

- Design: Retrospective cohort study.
- Participants: Singleton pregnancies between 1990 and 2004 in the Mid-Ohio Valley. Participants are part of the much larger C8 Health Project. Case-control sampling identified cases of term low birth weight (n=918) and a 10% sample of term >/= 2500 grams controls (n=3616).
- Exposure: PFOA exposure estimated retrospectively based on environmental, exposure and pharmacokinetic modeling in conjunction with birth records and geo identifiers of birth residence assumed for last 6 yrs. Geocodes determined public vs. private well water source; multiple imputations used for zip code only births (34%) to generate new exposure estimate. (Continuous and quintiles of exposure used in analysis). n=4,534.
- Outcomes: Birth weight (continuous and dichotomous; beta statistic, odds ratio), small for gestational age.
- Notes: Although excluded from analysis of PFOA and term low birth weight, the relationship between PFOA and pre-term birth was examined separately (findings: non-significant adjusted OR).

Table S18. Characteristics of Savitz et al. 2012b (Study 2).

Bias	Rating	Support for judgment
Recruitment	Low risk	The strategy for recruiting participants was consistent across study groups.
Blinding	Probably low risk	The authors did not discuss blinding but the study design prevents knowledge of exposure groups.
Confounding	Low risk	The authors adjusted for both maternal age and gestational age in their analysis.
Exposure assessment	Probably high risk	Exposure was retrospectively estimated using environmental, exposure and pharmacokinetic modeling with several assumptions, although moderate correlation between predicted vs. observed serum PFOA was reported. Modeling used lifetime residential history and exposure estimates were calibrated with serum levels measured after the study period.
Incomplete outcome data	Low risk	The study did not have incomplete outcome data.
Selective reporting	Low risk	The study is free of suggestion of selective outcome reporting. All of the study's specified outcomes were adequately reported.
Other bias	Low risk	No other potential biases are suspected.
Conflict of interest	Low risk	The authors report no conflict of interest, and associated funds and persons appear to be from government and/or academia only.

- Design: Retrospective cohort study.
- Participants: Singleton pregnancies between 1990 and 2004 in the Mid-Ohio Valley. Participants are part of the much larger C8 health Project.
- Exposure: PFOA exposure estimated retrospectively based on environmental, exposure and pharmacokinetic modeling (same as Study 1) in conjunction with birth records linked to self-reported births from C8 Health Project which provided lifetime residential history. Three methods of calculating exposure estimates were used: uncalibrated model estimates; Bayesian time-dependent calibration that used 2005-2006 serum concentration to update estimates; and traditional calibration that used 2005-2006 serum concentrations to update estimates and assumed that "higher than expected" serum concentration reflects lifetime (continuous and quintiles of exposure used in analysis). n=4,142.

- Outcomes: Birth weight (continuous and dichotomous; beta statistic, odds ratio), small for gestational age.
- Notes: Although excluded from analysis of PFOA and term low birth weight, the relationship between PFOA and pre-term birth was examined separately (findings: some evidence of elevated adjusted odds).

Table S19. Characteristics of Stein et al. 2009.

Bias	Rating	Support for judgment
Recruitment	Low risk	The strategy for recruiting participants was consistent across study groups.
Blinding	High risk	The outcome data were self-reported and the participants were aware of being exposed to PFOA because they were part of a publicized study that was funded by a lawsuit.
Confounding	Probably high risk	The authors restricted the analysis of birth weight to term births but did not account for gestational age in the analysis of term birth weight.
Exposure assessment	Probably high risk	Serum levels measured up to 5 years after birth were used to estimate exposure. The description of laboratory analysis also lacks detail.
Incomplete outcome data	Probably low risk	One participant was missing from analysis without explanation, but not likely to affect results.
Selective reporting	Low risk	The study is free of suggestion of selective outcome reporting. All of the study's specified outcomes were adequately reported.
Other bias	Low risk	No other potential biases are suspected.
Conflict of interest	Low risk	The authors report no conflict of interest, and associated funds and persons appear to be from government and/or academia only.

- Design: Cross-sectional study; retrospective account of outcome data.
- Participants: Pregnancies among female C8 Health Project participants between 2000 and 2006 with serum PFC measurements.
- Exposure: PFOA concentrations in maternal serum collected up to five years after birth (continuous and percentiles). n=1589 live births.
- Outcomes: Birth weight (dichotomous data; odds ratio).
- Notes: Although excluded from analysis of PFOA and term low birth weight, the relationship between PFOA and pre-term birth was examined separately (findings: non-significant adjusted OR).

Table S20. Characteristics of Washino et al. 2009.

Bias	Rating	Support for judgment
Recruitment	Low risk	The strategy for recruiting participants was consistent across study groups.
Blinding	Probably low risk	The authors did not discuss blinding but the study design prevents knowledge of exposure groups.
Confounding	Low risk	The authors adjusted for both maternal age and gestational age in their analysis.
Exposure assessment	Low risk	The exposure assessment methods were robust and included a detailed description of QA/QC.
Incomplete outcome data	Low risk	The study did not have incomplete outcome data.
Selective reporting	Low risk	The study is free of suggestion of selective outcome reporting. All of the study's specified outcomes were adequately reported.
Other bias	Low risk	No other potential biases are suspected.
Conflict of interest	Low risk	The authors report no conflict of interest, and associated funds and persons appear to be from government and/or academia only.

- Design: Prospective cohort study; cross-sectional exposure.
- Participants: Pregnant women who delivered at Sapporo Toho Hospital, Japan between July 2002 and October 2005.
- Exposure: PFOA concentrations in maternal serum collected after 2nd trimester (continuous). n=428.
- Outcomes: Birth weight (continuous data; beta statistic), length, chest circumference, head circumference.
- Notes: Received raw individual data from authors.

Table S21. Characteristics of Whitworth et al. 2012.

Bias	Rating	Support for judgment
Recruitment	Probably low risk	The study lacks description of randomization and the study population is divided into two categories (random, subfecund) from a previous study, but otherwise no reason to suspect there were substantial differences between comparison groups other than PFOA exposure.
Blinding	Probably low risk	The authors described blinding only for the QA/QC but the study design prevents knowledge of exposure groups.
Confounding	Low risk	The authors adjusted for both maternal age and gestational age in their analysis.
Exposure assessment	Probably low risk	Incomplete description of the quality assurance and control aspects of the exposure measurements as outlined in the protocol, but otherwise no problems relating to exposure misclassification were detected.
Incomplete outcome data	Low risk	Incomplete outcome data were adequately addressed.
Selective reporting	Low risk	The study is free of suggestion of selective outcome reporting. All of the study's specified outcomes were adequately reported.
Other bias	Low risk	No other potential biases are suspected.
Conflict of interest	Low risk	The authors report no conflict of interest, and associated funds and persons appear to be from government and/or academia only.

- Design: Nested case-control study.
- Participants: Women enrolled in Norwegian Mother and Child Cohort Study between 2003 and 2004 selected for a prior case-based study of PFCs and subfecundity.
- Exposure: PFOA concentrations in maternal serum collected at the time of enrollment (continuous and quartile). n=849.
- Outcomes: Birth weight (continuous data; beta statistic), small for gestational age, large for gestational age.
- Notes: Although excluded from analysis of PFOA and term low birth weight, the relationship between PFOA and pre-term birth was examined separately (findings: non-significant adjusted OR).

Table S22. Meta-analysis for association between PFOA exposure and birth length (cm).

Study	n	Effect estimate (95% CI)	%Weight of study in meta-analysis
Apelberg et al. 2007	293	-0.062 (-0.35, 0.23)	1.5
Fei et al. 2008	1347	-0.069 (-0.11, -0.03)	62.6
Washino et al. 2009	428	0.041 (-0.16, 0.25)	2.9
Maisonet et al. 2012	356	-0.090 (-0.19, 0.008)	12.9
Chen et al. 2012	429	-0.009 (-0.09, 0.07)	20.1
Overall effect	2853	-0.056 (-0.09, -0.02)	100

Der Simonian-Laird random effects model. p-value for heterogeneity = 0.55; I^2 = 0%

Adjusted estimates. Apelberg et al.: maternal age, gestational age; Chen et al.: maternal age, gestational age; Fei et al.: maternal age, gestational age, quadratic gestational age, infant sex, socio-occupational status, parity, smoking, pre-pregnancy body mass index, gestational week at blood draw; Maisonet et al.: smoking, pre-pregnancy body mass index, education, previous live birth, gestational age; Washino et al.: maternal age, gestational age.

Table S23. Meta-analysis for association between PFOA exposure and ponderal index.

**Study**	**n**	**Effect estimate (95% CI)**	**%Weight of study in meta-analysis**
Apelberg et al. 2007	293	-0.043 (-0.079, -0.006)	13.6
Washino et al. 2009	428	-0.031 (-0.072, 0.009)	11.4
Maisonet et al. 2012	360	0.005 (-0.008, 0.018)	35.3
Chen et al. 2012	429	-0.007 (-0.017, 0.003)	39.7
Overall effect	1510	-0.010 (-0.026, 0.005)	100

Der Simonian-Laird random effects model. p-value for heterogeneity = 0.045; I^2 = 63%

Adjusted estimates. Apelberg et al.: maternal age, gestational age; Chen et al.: maternal age, gestational age; Maisonet et al.: pre-pregnancy body mass index, previous live birth, gestational age at blood draw; Washino et al.: maternal age, gestational age.

Table S24. Meta-analysis for association between PFOA exposure and head circumference (cm).

Study	n	Effect estimate (95% CI)	% Weight of study in meta-analysis
Apelberg et al. 2007	293	-0.21 (-0.41, -0.02)	4.3
Fei et al. 2008	1347	-0.03 (-0.06, 0.004)	56.4
Chen et al. 2012	429	-0.01 (-0.07, 0.05)	32.3
Washino et al. 2009	428	-0.07 (-0.22, 0.08)	7.1
Overall effect	2497	-0.03 (-0.08, 0.007)	100

Der Simonian-Laird random effects model. p-value for heterogeneity = 0.26; I^2 = 26%

Adjusted estimates. Apelberg et al.: maternal age, gestational age; Chen et al.: maternal age, gestational age; Fei et al.: maternal age, gestational age, quadratic gestational age, infant sex, socio-occupational status, parity, smoking, pre-pregnancy body mass index, gestational week at blood draw; Washino et al.: maternal age, gestational age.

References

Apelberg BJ, Witter FR, Herbstman JB, Calafat AM, Halden RU, Needham LL, et al. 2007. Cord serum concentrations of perfluorooctane sulfonate (PFOS) and perfluorooctanoate (PFOA) in relation to weight and size at birth. Environ Health Perspect 115:1670-1676.

Arbuckle TE, Kubwabo C, Walker M, Davis K, Lalonde K, Kosarac I, et al. 2012. Umbilical cord blood levels of perfluoroalkyl acids and polybrominated flame retardants. Int J Hyg Environ Health 216:184-194.

Boyles AL, Harris SF, Rooney AA, Thayer KA. 2011. Forest plot viewer: A new graphing tool. Epidemiology 22:746-747.

Chen MH, Ha EH, Wen TW, Su YN, Lien GW, Chen CY, et al. 2012. Perfluorinated compounds in umbilical cord blood and adverse birth outcomes. PLoS One 7:e42474.

Fei C, McLaughlin JK, Tarone RE, Olsen J. 2007. Perfluorinated chemicals and fetal growth: A study within the danish national birth cohort. Environ Health Perspect 115:1677.

Fei C, McLaughlin JK, Tarone RE, Olsen J. 2008. Fetal growth indicators and perfluorinated chemicals: A study in the danish national birth cohort. Am J Epidemiol 168:66-72.

Fromme H, Mosch C, Morovitz M, Alba-Alejandre I, Boehmer S, Kiranoglu M, et al. 2010. Pre- and postnatal exposure to perfluorinated compounds (PFCs). Environmental science & technology 44:7123-7129.

Goldman LRA, B.J.; Herbstman, J.B.; Witter, .FR.; Calafat, A.; Halden, R.I.J. 2012. Reanalysis. (Johnson PI, ed). San Francisco, CA.

Halldorsson TI, Rytter D, Haug LS, Bech BH, Danielsen I, Becher G, et al. 2012. Prenatal exposure to perfluorooctanoate and risk of overweight at 20 years of age: A prospective cohort study. Environ Health Perspect 120:668.

Hamm MP, Cherry NM, Chan E, Martin JW, Burstyn I. 2010. Maternal exposure to perfluorinated acids and fetal growth. Journal of exposure science & environmental epidemiology 20:589-597.

Kim S, Choi K, Ji K, Seo J, Kho Y, Park J, et al. 2011. Trans-placental transfer of thirteen perfluorinated compounds and relations with fetal thyroid hormones. Environ Sci Technol 45:7465-7472.

Kim SK, Lee KT, Kang CS, Tao L, Kannan K, Kim KR, et al. 2011. Distribution of perfluorochemicals between sera and milk from the same mothers and implications for prenatal and postnatal exposures. Environmental Pollution 159:169-174.

Maisonet M, Terrell ML, McGeehin MA, Christensen KY, Holmes A, Calafat AM, et al. 2012. Maternal concentrations of polyfluoroalkyl compounds during pregnancy and fetal and postnatal growth in british girls. Environ Health Perspect 120:1432-1437.

Monroy R, Morrison K, Teo K, Atkinson S, Kubwabo C, Stewart B, et al. 2008. Serum levels of perfluoroalkyl compounds in human maternal and umbilical cord blood samples. Environ Res 108:56-62.

Nolan LA, Nolan JM, Shofer FS, Rodway NV, Emmett EA. 2009. The relationship between birth weight, gestational age and perfluorooctanoic acid (PFOA)-contaminated public drinking water. Reprod Toxicol 27:231-238.

Savitz DA, Stein CR, Bartell SM, Elston B, Gong J, Shin HM, et al. 2012a. Perfluorooctanoic acid exposure and pregnancy outcome in a highly exposed community. Epidemiology 23:386-392.

Savitz DA, Stein CR, Elston B, Wellenius GA, Bartell SM, Shin HM, et al. 2012b. Relationship of perfluorooctanoic acid exposure to pregnancy outcome based on birth records in the mid-Ohio valley. Environ Health Perspect 120:1201-1207.

Stein CR, Savitz DA, Dougan M. 2009. Serum levels of perfluorooctanoic acid and perfluorooctane sulfonate and pregnancy outcome. Am J Epidemiol 170:837-846.

Washino N, Saijo Y, Sasaki S, Kato S, Ban S, Konishi K, et al. 2009. Correlations between prenatal exposure to perfluorinated chemicals and reduced fetal growth. Environ Health Perspect 117:660-667.

Whitworth KW, Haug LS, Baird DD, Becher G, Hoppin JA, Skjaerven R, et al. 2012. Perfluorinated compounds in relation to birth weight in the norwegian mother and child cohort study. Am J Epidemiol 175:1209-1216.

Instructions for Making Risk of Bias Determinations

1. Was the strategy for recruiting participants consistent across study groups?

Criteria for a judgment of 'YES' (i.e. low risk of bias):

Protocols for recruitment and inclusion/exclusion criteria were applied similarly across study groups, and any one of the following:

- Study participants were recruited from the same population at the same time frame; or
- Study participants were not all recruited from the same population, but proportions of participants from each population in each study group are uniform

Criteria for the judgment of 'PROBABLY YES' (i.e. probably low risk of bias):

There is insufficient information about participant selection to permit a judgment of 'YES', but there is indirect evidence that suggests that participant recruitment and inclusion/exclusion criteria was consistent, as described by the criteria for a judgment of 'YES'.

Criteria for the judgment of 'NO' (i.e. high risk of bias):

Any one of the following:

- Protocols for recruitment or inclusion/exclusion criteria were applied differently across study groups; or
- Study participants were recruited at different time frames; or
- Study participants were recruited from different populations and proportions of participants from each population in each study group are not uniform

Criteria for the judgment of 'PROBABLY NO' (i.e. probably high risk of bias):

There is insufficient information about participant selection to permit a judgment of 'NO', but there is indirect evidence that suggests that participant recruitment or inclusion/exclusion criteria was inconsistent, as described by the criteria for a judgment of 'NO'.

Criteria for the judgment of 'NOT APPLICABLE' (risk of bias domain is not applicable to study):

There is evidence that participant selection is not an element of study design capable of introducing risk of bias in the study.

2. Was knowledge of the exposure groups adequately prevented during the study?

Criteria for a judgment of 'YES' (i.e. low risk of bias):

Any one of the following:

- No blinding, but the review authors judge that the outcome and the outcome measurement are not likely to be influenced by lack of blinding; or
- Blinding of key study personnel ensured, and unlikely that the blinding could have been broken; or
- Some key study personnel were not blinded, but outcome assessment was blinded and the non-blinding of others unlikely to introduce bias.

Criteria for the judgment of 'PROBABLY YES' (i.e. probably low risk of bias):

There is insufficient information about blinding to permit a judgment of 'YES', but there is indirect evidence that suggests the study was adequately blinded, as described by the criteria for a judgment of 'YES'.

Criteria for the judgment of 'NO' (i.e. high risk of bias):

Any one of the following:

- No blinding or incomplete blinding, and the outcome or outcome measurement is likely to be influenced by lack of blinding; or
- Blinding of key study personnel attempted, but likely that the blinding could have been broken; or
- Some key study personnel were not blinded, and the non-blinding of others likely to introduce bias.

Criteria for the judgment of 'PROBABLY NO' (i.e. probably high risk of bias):

There is insufficient information about blinding to permit a judgment of 'NO', but there is indirect evidence that suggests the study was not adequately blinded, as described by the criteria for a judgment of 'NO'.

Criteria for the judgment of 'NOT APPLICABLE' (risk of bias domain is not applicable to study):

There is evidence that blinding is not an element of study design capable of introducing risk of bias in the study.

3. Were exposure assessment methods robust?

Criteria for a judgment of 'YES' (i.e. low risk of bias):

The reviewers judge that there is low risk of exposure misclassification and any one of the following:

- There is high confidence in the accuracy of the exposure assessment methods; or
- Less-established or less direct exposure measurements are validated against well-established or direct methods

AND if applicable, appropriate QA/QC for methods are described and are satisfactory, with at least three of the following items reported, or at least two of the following items reported plus evidence of satisfactory performance in a high quality inter-laboratory comparison:

Limit of detection or quantification; standards recovery; measure of repeatability; investigation and prevention of blanks contamination.

Criteria for the judgment of 'PROBABLY YES' (i.e. probably low risk of bias):

There is insufficient information about the exposure assessment methods to permit a judgment of 'YES', but there is indirect evidence that suggests that methods were robust, as described by the criteria for a judgment of 'YES'. Studies only reporting that the QA/QC items above were satisfactory but not reporting all of the actual numbers may receive a judgment of "probably yes."

Criteria for the judgment of 'NO' (i.e. high risk of bias):

The reviewers judge that there is high risk of exposure misclassification and any one of the following:

- There is low confidence in the accuracy of the exposure assessment methods; or

- Less-established or less direct exposure measurements are not validated and are suspected to introduce bias that impacts the outcome assessment (example: participants are asked to report exposure status retrospectively, subject to recall bias)
- Uncertain how exposure information was obtained

Criteria for the judgment of 'PROBABLY NO' (i.e. probably high risk of bias):

There is insufficient information about the exposure assessment methods to permit a judgment of 'NO', but there is indirect evidence that suggests that methods were not robust, as described by the criteria for a judgment of 'NO'.

Criteria for the judgment of 'NOT APPLICABLE' (risk of bias domain is not applicable to study):

There is evidence that exposure assessment is not an element of study design capable of introducing risk of bias in the study.

4. Was confounding adequately addressed?

Criteria for a judgment of 'YES' (i.e. low risk of bias):

The study accounted for (i.e., matched, stratified, multivariate analysis or otherwise statistically controlled for) important potential confounders, or reported that potential confounders were evaluated and omitted because inclusion did not substantially affect the results. The determination of specific confounders may be informed by the data, including the studies included in the review.

Criteria for the judgment of 'PROBABLY YES' (i.e. probably low risk of bias):

The study accounted for most but not all of the important potential confounders AND this lack of accounting is not expected to introduce substantial bias.

Criteria for the judgment of 'NO' (i.e. high risk of bias):

The study did not account for or evaluate important potential confounders.

Criteria for the judgment of 'PROBABLY NO' (i.e. probably high risk of bias):

The study accounted for some but not all of the important potential confounders

AND this lack of accounting may have introduced substantial bias.

5. Were incomplete outcome data adequately addressed?

Criteria for a judgment of 'YES' (i.e. low risk of bias):

Participants were followed long enough to obtain outcome measurements and any one of the following:

- No missing outcome data; or
- Reasons for missing outcome data unlikely to be related to true outcome (for survival data, censoring unlikely to be introducing bias); or
- Missing outcome data balanced in numbers across exposure groups, with similar reasons for missing data across groups; or
- For dichotomous outcome data, the proportion of missing outcomes compared with observed event risk not enough to have a biologically relevant impact on the intervention effect estimate; or
- For continuous outcome data, plausible effect size (difference in means or standardized difference in means) among missing outcomes not enough to have a biologically relevant impact on observed effect size; or
- Missing data have been imputed using appropriate methods

Criteria for the judgment of 'PROBABLY YES' (i.e. probably low risk of bias):

There is insufficient information about incomplete outcome data to permit a judgment of 'YES', but there is indirect evidence that suggests incomplete outcome data was adequately addressed, as described by the criteria for a judgment of 'YES'.

Criteria for the judgment of 'NO' (i.e. high risk of bias):

Participants were not followed long enough to obtain outcome measurements OR any one of the following:

- Reason for missing outcome data likely to be related to true outcome, with either imbalance in numbers or reasons for missing data across exposure groups; or

- For dichotomous outcome data, the proportion of missing outcomes compared with observed event risk enough to induce biologically relevant bias in intervention effect estimate; or
- For continuous outcome data, plausible effect size (difference in means or standardized difference in means) among missing outcomes enough to induce biologically relevant bias in observed effect size; or
- Potentially inappropriate application of imputation.

Criteria for the judgment of 'PROBABLY NO' (i.e. probably high risk of bias):

There is insufficient information about incomplete outcome data to permit a judgment of 'NO', but there is indirect evidence that suggests incomplete outcome data was not adequately addressed, as described by the criteria for a judgment of 'NO'.

Criteria for the judgment of 'NOT APPLICABLE' (risk of bias domain is not applicable to study):

There is evidence that incomplete outcome data is not capable of introducing risk of bias in the study.

6. Are reports of the study free of suggestion of selective outcome reporting?

Criteria for a judgment of 'YES' (i.e. low risk of bias):

All of the study's pre-specified (primary and secondary) outcomes outlined in the protocol, methods, abstract, and/or introduction that are of interest in the review have been reported in the pre-specified way.

Criteria for the judgment of 'PROBABLY YES' (i.e. probably low risk of bias):

There is insufficient information about selective outcome reporting to permit a judgment of 'YES', but there is indirect evidence that suggests the study was free of selective reporting, as described by the criteria for a judgment of 'YES'.

Criteria for the judgment of 'NO' (i.e. high risk of bias):

Any one of the following:

- Not all of the study's pre-specified primary outcomes (as outlined in the protocol, methods, abstract, and/or introduction) have been reported; or
- One or more primary outcomes is reported using measurements, analysis methods or subsets of the data (e.g. subscales) that were not pre-specified; or
- One or more reported primary outcomes were not pre-specified (unless clear justification for their reporting is provided, such as an unexpected effect); or

One or more outcomes of interest are reported incompletely

Criteria for the judgment of 'PROBABLY NO' (i.e. probably high risk of bias):

There is insufficient information about selective outcome reporting to permit a judgment of 'NO', but there is indirect evidence that suggests the study was not free of selective reporting, as described by the criteria for a judgment of 'NO'.

Criteria for the judgment of 'NOT APPLICABLE' (risk of bias domain is not applicable to study):

There is evidence that selective outcome reporting is not capable of introducing risk of bias in the study.

7. Was the study apparently free of other problems that could put it at a risk of bias?

Criteria for a judgment of 'YES' (i.e. low risk of bias):

The study appears to be free of other sources of bias.

Criteria for the judgment of 'PROBABLY YES' (i.e. probably low risk of bias):

There is insufficient information to permit a judgment of 'YES', but there is indirect evidence that suggests the study was free of other threats to validity.

Criteria for the judgment of 'NO' (i.e. high risk of bias):

There is at least one important risk of bias. For example, the study:

- Had a potential source of bias related to the specific study design used; or
- Stopped early due to some data-dependent process (including a formal-stopping rule); or
- Had extreme imbalance of characteristics among exposure groups; or

- Had differential surveillance for outcome between exposure groups or between exposed/unexposed groups
- The conduct of the study is affected by interim results (e.g. recruiting additional participants from a subgroup showing greater or lesser effect); or
- An insensitive instrument is used to measure outcomes (which can lead to under-estimation of both beneficial and harmful effects); or
- Selective reporting of subgroups; or
- Has been claimed to have been fraudulent; or
- Had some other problem

Criteria for the judgment of 'PROBABLY NO' (i.e. probably high risk of bias):

There is insufficient information to permit a judgment of 'NO', but there is indirect evidence that suggests the study was not free of other threats to validity, as described by the criteria for a judgment of 'NO'.

Criteria for the judgment of 'NOT APPLICABLE' (risk of bias domain is not applicable to study):

There is evidence that other potential threats to validity are not capable of introducing risk of bias in the study.

8. Was the study free of support from a company, study author, or other entity having a financial interest in any of the exposures studied?

Criteria for a judgment of 'YES' (i.e. low risk of bias):

The study did not receive support from a company, study author, or other entity having a financial interest in the outcome of the study. Examples include the following:

- Funding source is limited to government, non-profit organizations, or academic grants funded by government, foundations and/or non-profit organizations;
- Chemicals or other treatment used in study were purchased from a supplier;
- Company affiliated staff are not mentioned in the acknowledgements section;
- Authors were not employees of a company with a financial interest in the outcome of the study;

- Company with a financial interest in the outcome of the study was not involved in the design, conduct, analysis, or reporting of the study and authors had complete access to the data;
- Study authors make a claim denying conflicts of interest;
- Study authors are unaffiliated with companies with financial interest, and there is no reason to believe a conflict of interest exists;
- All study authors are affiliated with a government agency (are prohibited from involvement in projects for which there is a conflict of interest or an appearance of conflict of interest).

Criteria for the judgment of 'PROBABLY YES' (i.e. probably low risk of bias):

There is insufficient information to permit a judgment of 'YES', but there is indirect evidence that suggests the study was free of support from a company, study author, or other entity having a financial interest in the outcome of the study, as described by the criteria for a judgment of 'YES'.

Criteria for the judgment of 'NO' (i.e. high risk of bias):

The study received support from a company, study author, or other entity having a financial interest in the outcome of the study. Examples of support include:

- Research funds;
- Chemicals provided at no cost;
- Writing services;
- Author/staff from study was employee or otherwise affiliated with company with financial interest;
- Company limited author access to the data;
- Company was involved in the design, conduct, analysis, or reporting of the study;
- Study authors claim a conflict of interest

Criteria for the judgment of 'PROBABLY NO' (i.e. probably high risk of bias):

There is insufficient information to permit a judgment of 'NO', but there is indirect evidence that suggests the study was not free of support from a company, study author, or other entity having a financial interest in the outcome of the study, as described by the criteria for a judgment of 'NO'.

Criteria for the judgment of 'NOT APPLICABLE' (risk of bias domain is not applicable to study):

There is evidence that conflicts of interest are not capable of introducing risk of bias in the study.

Figure legend

Figure S1. Summary of data extracted from all studies of PFOA exposure with a dichotomous outcome of birth weight. Low birth weight is defined as less than 2500 grams. The PFOA increase is the exposure contrast being compared in each study. Square markers represent data where there was an exposure gradient that can be evaluated when considering dose-response in upgrading the quality of the evidence (Table 6). Savitz et al. 2012b paper presented additional alternative estimates not included in Figure S1 due to space limitations. Nolan et al. 2009, Savitz et al. 2012a, Savitz et al. 2012b and Stein et al. 2009 were conducted in the same geographical area and participants may overlap.

Covariate abbreviations from each study: ga=gestational age; ma=maternal age; bmi=body mass index; par=parity; smk=smoking status; sex=infant gender; ht=maternal height; wtg=maternal weight gain during pregnancy; dia=diabetes; hyp=hypertension; cot=serum cotinine; edu=maternal education level; delmode= delivery mode; SES=socioeconomic status; gabd=gestational age at blood draw; PFOS=serum perfluorooctane ulfonic acid; grav=gravidity; mwt=maternal prepregnancy weight; exposyr=year of exposure estimate; state=state of residence; bsp=blood sampling period. Figure S1 was created using Meta Data Viewer (Boyles 2011).

Summary of All Studies with Odds Ratios for Low Birth Weight

Study	*PFOA increase*	*Comparison (ng/mL)*	*Covariates*
Chen et al 2012	ln ng/mL	geomean(stdev)=1.84(2.23)	ga, ma, bmi, par, cot, sex, edu, delivmode
Fei et al 2007	first to second quartile	</=3.91 to 3.91-5.20 ng/ml	ga, ma, bmi, par, smk, sex, SES, gabd
Fei et al 2007	first to third quartile	</=3.91 to 5.21-6.96 ng/ml	ga, ma, bmi, par, smk, sex, SES, gabd
Fei et al 2007	first to fourth quartile	</=3.91 to >/= 6.97 ng/ml	ga, ma, bmi, par, smk, sex, SES, gabd
Nolan et al 2009	low to mid exposure	na	ga, ga2, ga3, ma, race, sex, SES
Nolan et al 2009	low to high exposure	na	ga, ga2, ga3, ma, race, sex, SES
Savitz et al 2012a	25th to 75th IQR (lnPFOA)	2.19	ma, par, smk, edu, exposyr
Savitz et al 2012a	100 ng/mL	100 ng/mL	ma, par, smk, edu, exposyr
Savitz et al 2012a	<40th to 40-<60th percentile	3.9-<6.8 to 6.8-<16.6	ma, par, smk, edu, exposyr
Savitz et al 2012a	<40th to 60-<80th percentile	3.9-<6.8 to 16.6-<63.1	ma, par, smk, edu, exposyr
Savitz et al 2012a	<40th to >/= 80th percentile	3.9-<6.8 to 63.1-934.3	ma, par, smk, edu, exposyr
Savitz et al 2012b Study II-B	25th to 75th IQR (lnPFOA)	1.92	ga, ma, par, edu, smk, exposyr, state
Savitz et al 2012b Study II-B	100 ng/ml	100 ng/mL	ga, ma, par, edu, smk, exposyr, state
Savitz et al 2012b Study II-B	1st/2nd quintile to 3rd quintile	3.9 - <8.9 to 8.9 - <19.6	ga, ma, par, edu, smk, exposyr, state
Savitz et al 2012b Study II-B	1st/2nd quintile to 4th quintile	3.9 - <8.9 to 19.6 - 53.1	ga, ma, par, edu, smk, exposyr, state
Savitz et al 2012b Study II-B	1st/2nd quintile to 5th quintile	3.9 - <8.9 to 53.1 -1897.0	ga, ma, par, edu, smk, exposyr, state
Stein et al 2009	25th to 75th percentile	10.3-49.8	ma, par, smk, edu, PFOS
Stein et al 2009	<50th to >/= 50th percentile	0.25 - <21.3 to 21.3-894.4	ma, par, smk, edu, PFOS
Stein et al 2009	<50th to 50th - <75th percentile	0.25 - <21.3 to 21.3 - <50	ma, par, smk, edu, PFOS
Stein et al 2009	<50th to 75th - 90th percentile	0.25 - <21.3 to 50 - <120.6	ma, par, smk, edu, PFOS
Stein et al 2009	<50th to >90th percentile	0.25 - <21.3 to 120.6 - 894.4	ma, par, smk, edu, PFOS

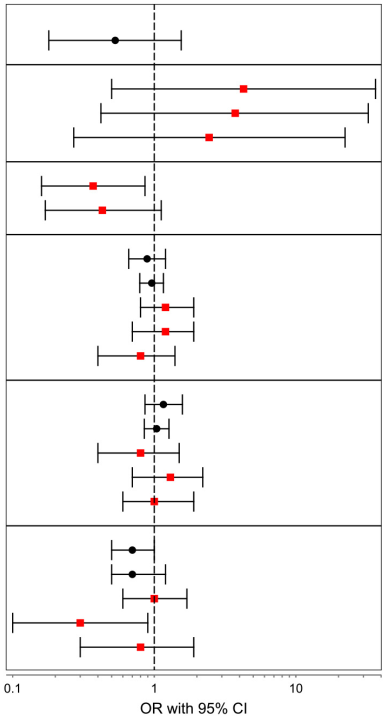

● beta estimate ■ beta estimate that can be used to evaluate dose-response

Figure S1
